# Causal associations between constipation and pan-cancer: a bidirectional Mendelian randomization study

**DOI:** 10.3389/fonc.2024.1428003

**Published:** 2024-09-13

**Authors:** Yongze Dang, Xinyu He, Xiaoxiao Liu, Yuchen Wang, Shangyi Geng, Yutong Cheng, Hongbing Ma, Xixi Zhao

**Affiliations:** ^1^ Department of Radiation Oncology, The Second Affiliated Hospital of Xi’an Jiaotong University, Xi’an, China; ^2^ Department of Gastroenterology, The Second Affiliated Hospital of Xi’an Jiaotong University, Xi’an, China

**Keywords:** constipation, pan cancer, Mendelian Randomization, GWAS, causality

## Abstract

**Objective:**

Observational studies have suggested a potential association between constipation and several cancers. However, the causal relationship between constipation and cancer remains unclear. The purpose of this study is to explore the potential causal relationship between constipation and pan-cancer using Mendelian Randomization (MR) methods.

**Methods:**

We performed a bidirectional MR analysis using publicly available summary data from Genome-Wide Association Studies (GWAS) statistics. The Inverse Variance Weighted (IVW) method was used as the main analysis method. We also used four MR methods: MR-Egger, Weighted Median, MR-PRESSO and MR.RAPS. Simultaneously, MR-Egger regression, Cochran’s Q test and MR-PRESSO Global test were used to estimate the pleiotropy and heterogeneity of SNPs. In addition, we performed “leave-one-out” analyses” to avoid bias caused by horizontal pleiotropy of individual SNPs.

**Results:**

MR analysis revealed a potential causal association between constipation and the risk of colorectal cancer (CRC) [IVW (OR= 1.0021 (1.0003, 1.0039), P= 0.0234)], lung cancer (LC) [IVW (OR=1.0955 (1.0134, 1.1843), P=0.0218)], Oral cavity and pharyngeal cancer (OPC) [IVW (OR=1.4068 (1.0070, 1.9652), P=0.0454)], and Pancreatic cancer (PC) [IVW (OR=1.5580 (1.0659, 2.2773), P=0.0221)]. In addition, we explored causal relationships between constipation and 12 other types of cancers, including gastric cancer, esophageal cancer, skin melanoma and so on. All five methods yielded no evidence of a causal association between constipation and the risk of these cancer types. In the reverse MR analysis, there was no evidence of a causal association between cancer and the risk of constipation for all five methods.

**Conclusion:**

Our bidirectional MR study suggests a potential relationship between constipation and an increased risk of CRC, LC OPC and PC. The underlying mechanisms behind these associations will need to be explored in future experimental studies.

## Introduction

Cancer is a serious global health issue, causing nearly 10 million deaths each year (1). Despite significant advancements in cancer treatment, lagging progress in cancer prevention continues to contribute to rising incidence rates across various cancers (2). Identifying modifiable risk factors for cancer is crucial for the development of effective prevention strategies (3).

Constipation is a common gastrointestinal issue characterized by difficult bowel movements, decreased frequency of defecation, and hard stools (4, 5). In recent years, increasing attention has been paid to the relationship between constipation and cancer risk. The potential mechanisms mainly include the following aspects: Constipation is closely associated with changes and imbalances in the gut microbiota (6, 7). The intestinal microbiota may play a significant role in the development and progression of cancer (8, 9). Additionally, constipation can cause chronic inflammatory responses in the intestinal mucosa (10). Chronic inflammation is a significant factor in cancer development, as prolonged inflammatory states can lead to cellular mutations and carcinogenesis (11). Moreover, long-term constipation may result in the retention and reabsorption of toxins and carcinogens within the body, such as bile acids, which can increase the risk of CRC (12). Therefore, we hypothesize that there may be an association between constipation and cancer.

Previous epidemiological studies have suggested an association between constipation and certain types of cancer. Several cohort and case-control studies have investigated the link between constipation and CRC, but the results remain highly controversial (13–16). Furthermore, some cohort studies have indicated a potential association between constipation and non-gastrointestinal cancers, such as ovarian and breast cancer (17, 18). However, evidence for the association between constipation and other cancers is inconsistent or lacking. This suggests the need for a pan-cancer analysis to systematically assess the association between constipation and the risk of cancer.

Furthermore, potential unmeasured confounders or reverse causation exist in observational studies, limiting their ability to establish a causal relationship between constipation and cancer. Therefore, we employed MR analysis to investigate the potential causal relationship between constipation and cancer. MR analysis is a statistical strategy based on genome-wide association analysis, utilizing genetic variations to determine whether a causal effect exists between risk factors and outcomes (19). This approach minimizes bias from confounding and reverse causation, providing a more accurate estimation of the effect of exposure on outcomes (20). Our study used a bidirectional two-sample MR method to analyze the effect of constipation on cancer risk and the interaction effect of cancer risk on the occurrence of constipation. By elucidating these bidirectional relationships, we aim to provide robust evidence to aid in understanding the interactions between constipation and cancer, offering guidance for clinicians in devising prevention strategies of cancer prevention strategies.

## Materials and methods

### Study design

**Figure 1:**
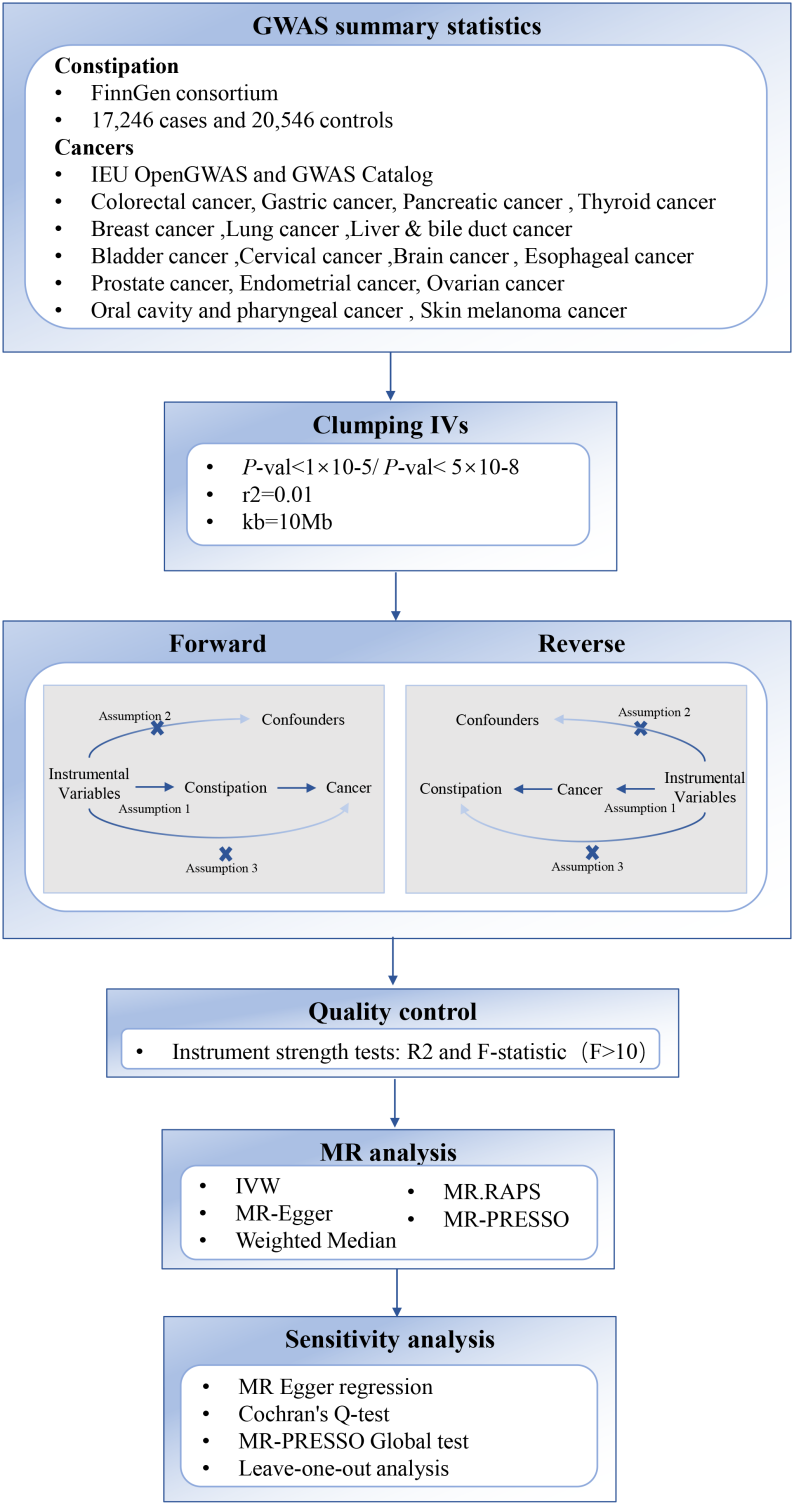
Overview of the two-sample MR study design used to investigate the causal association between Constipation and Cancer. SNP, Single Nucleotide Polymorphism; IVW, Inverse Variance Weighted; GWAS, genome-wide association study.

The overview of the study design is depicted in **Figure 1**. We conducted a bidirectional two-sample MR analysis on constipation and cancer using publicly available online data from the IEU OpenGWAS (https://gwas.mrcieu.ac.uk/) and GWAS Catalog (https://www.ebi.ac.uk/gwas). These databases have obtained ethical approvals and informed consent, obviating the need for additional explanations. Three fundamental assumptions must be satisfied during MR analysis. First, the relevance hypothesis: Instrumental variables (IVs) must be closely associated with constipation, with the F value considered a measure of this association. Second, the independence hypothesis: IVs and any potential confounding factors must be mutually independent. In other words, constipation IVs should not be related to other factors causally linked to cancer. Third, the exclusion-restriction hypothesis: IVs solely affect cancer through constipation.

### Data sources

GWAS summary statistics for constipation were obtained from the FinnGen consortium, including 17,246 cases and 20,546 controls. Genetic information associated with the following 13 types of malignant tumors can be found at the IEU OpenGWAS project (https://gwas.mrcieu.ac.uk/): colorectal cancer (5,657 cases/372,016 controls), oral cavity and pharyngeal cancer (2,497 cases/2,928 controls), esophageal cancer (740 cases/372,016 controls), skin melanoma (3,751 cases/372,016 controls), breast cancer (122,977 cases/105,974 controls), lung cancer (29,863 cases/55,586 controls), liver & bile duct cancer (350 cases/372,016 controls), endometrial cancer (12,906 cases/108,979 control), bladder cancer (1,279 cases/372,016 control), cervical cancer (563 cases/198,523 control), brain cancer (606 cases/372,016 control), ovarian cancer (1,218 cases/198,523 control), prostate cancer (79,148 cases/61,106 control). Data on genetic variations associated with gastric cancer (145 cases/456,203 controls), pancreatic cancer (587 cases/455,761 controls) and thyroid cancer (6,015 cases/1,333,754 controls) were taken from GWAS Catalog (https://www.ebi.ac.uk/gwas).

Only European populations were included in this study and there was no sample overlap in this MR study. Detailed information for constipation and cancer GWAS information is provided in **Supplementary Table 1**.

### Selection of genetic instrumental variables

To screen for eligible genetic IVs that satisfy the three core MR assumptions, we implemented a set of quality control techniques. Firstly, we selected independent Single Nucleotide Polymorphisms (SNPs) strongly associated with lung, breast, prostate cancer with a p-value of less than 5 × 10-8. We expanded the p-value threshold to 1 × 10-5 for sufficient instrumental variables for constipation and the remaining 13 cancers. Secondly, IVs were excluded based on linkage disequilibrium (LD) (r2≥0.01, kb > 10,000) (21). SNPs with palindromic structures were removed to avoid chain disambiguation problem (22). Additionally, we calculated the R2 statistic and F statistic for each SNP in the exposure. IVs with an F statistic less than 10 were considered weak instrumental variables and were excluded from MR analysis (23). The F-statistic of each SNP was calculated as the following formula:


$$\text{F}=\frac{R^{2}\times \left(N-2\right)}{1-R^{2}}$$

where R^2^ is the proportion of the variability of the exposure explained by each instrument and N is the sample size of the GWAS for the SNP- exposure association. To calculate R^2^ for each SNP we used the following formula:


$$R^{2}=\frac{2\times \beta^{2}\times EAF\times \left(1-EAF\right)}{2\times \beta^{2}\times EAF\times \left(1-EAF\right)+2\times S{E(\beta )}^{2}\times N\times EAF\times \left(1-EAF\right)}$$

where EAF is the effect allele frequency, β is the estimated genetic effect on exposure, N is the sample size of the GWAS for the SNP- exposure association and SE (β) is the standard error of the genetic effect (24).

Finally, we used PhenoScanner (http://www.phenoscanner.medschl.cam.ac.uk/) and GWAS Catalog(https://www.ebi.ac.uk/gwas) to examine whether the instrumental variables used were associated with potential confounding factors at a genome-wide significance (p < 5 × 10−8).

### Mendelian randomization analysis

In this study, we used the Inverse Variance Weighted (IVW) method as the primary analytic method to estimate the causal effect of exposure on outcomes. The IVW method combines Wald ratio estimates of the causal effects of different SNPs and provides a consistent estimate of the causal effect of exposure on outcomes. IVW can provide accurate estimates if all included SNPs are effective IVs (25). Additionally, four other MR methods were utilized: MR-Egger, Weighted Median, MR-PRESSO and MR.RAPS. MR-PRESSO is a method for the detection and correction of outliers in IVW linear regression (26). The Weighted Median method will provide an unbiased estimate of the causal effect in the presence of unbalanced horizontal pleiotropy even when up to 50% of SNPs are invalid IVs (27). The MR-Egger regression, based on the assumption of InSIDE, performs a weighted linear regression of the outcome coefficients on the exposure coefficients (28). The MR.RAPS method, robust to both systematic and idiosyncratic pleiotropy, provides reliable inference for MR analysis with numerous weak instruments by correcting for pleiotropy using robust adjusted profile scores (29).

All MR analyses were performed using the TwoSampleMR package (Version: 0.6.6), ggplot2 package (Version: 3.4.4), mr.raps package (Version: 0.2) and MRPRESSO package (Version: 1.0) in RStudio software (Version: 4.3.1). The test level α was 0.05 (p<0.05) and the difference was statistically significant.

### Sensitivity analysis

We used Cochran’s Q-test to estimate the heterogeneity of SNPs (28, 30). A p-value greater than 0.05 indicated no heterogeneity. In cases where heterogeneity was present, a random-effects IVW method was used to estimate the causal association. To evaluate pleiotropy, we used the intercept p-value obtained from the MR Egger regression and global test p-value of MR-PRESSO, with p > 0.05 indicating no potential pleiotropy of IVs (26). In addition, we performed leave-one-out analyses to avoid bias caused by horizontal pleiotropy of individual SNPs.

## Results

### SNPs data

In selecting the IVs, we strictly followed the above criteria. Consequently, 29 independent SNPs were selected as IVs for constipation from the total number of 16,380,466 SNPs with a p-value of less than 5 × 10-8. Additionally, We selected independent SNPs strongly associated with lung, breast, prostate cancer with a p-value of less than 5 × 10-8. We expanded the p-value threshold to 1 × 10-5 to ensure enough SNPs were available for MR analysis for other 13 malignant tumors. For detailed information, please refer to **Supplementary Table 2**. The F-statistic is also presented in the **Supplementary Table 2** and showed no evidence of weak instrumental bias. To further evaluate the stability of our study, the PhenoScanner and GWAS Catalog database was used to exclude SNPs associated with any potential confounders, of which one SNP (rs2867922) was excluded due to red cell distribution width in the forward MR analyses. Bidirectional MR analyses were performed for constipation and pan-cancer, and the all results of the analyses are presented in **Supplementary Table 3**. In addition, the number of IVs used in the MR analyses between constipation and the various types of cancer was not equal due to the results extracted from the different outcome datasets and the absence of the palindromic SNPs.

### Constipation and LC

**Figure 2:**
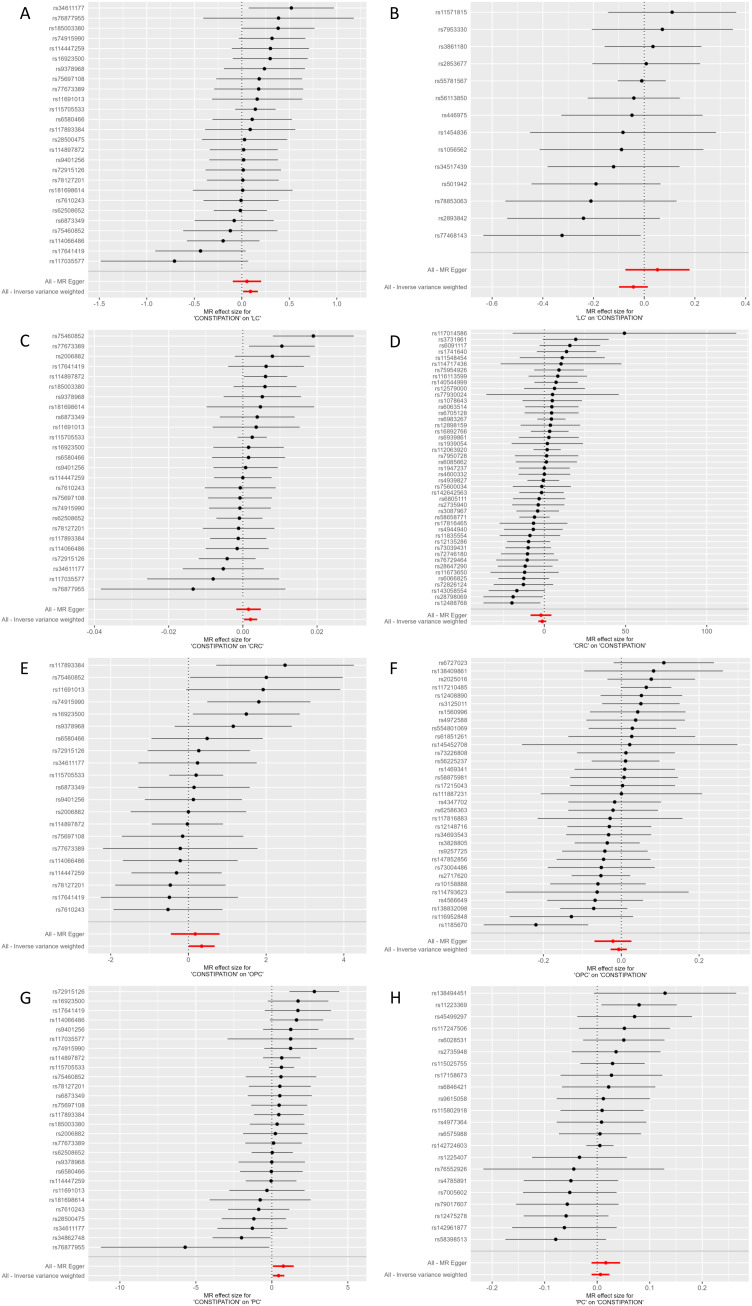
Forest plot of MR effect of the causal relationship between Constipation and significant Cancer **(A)** Constipation on LC; **(B)** LC on Constipation; **(C)** Constipation on CRC; **(D)** CRC on Constipation; **(E)** Constipation on OPC; **(F)** OPC on Constipation; **(G)** Constipation on PC; **(F)** PC on Constipation; MR, Mendelian Randomization; CRC, Colorectal cancer; LC, Lung cancer; OPC, Oral cavity and pharyngeal cancer; PC, Pancreatic cancer.

**Figure 3:**
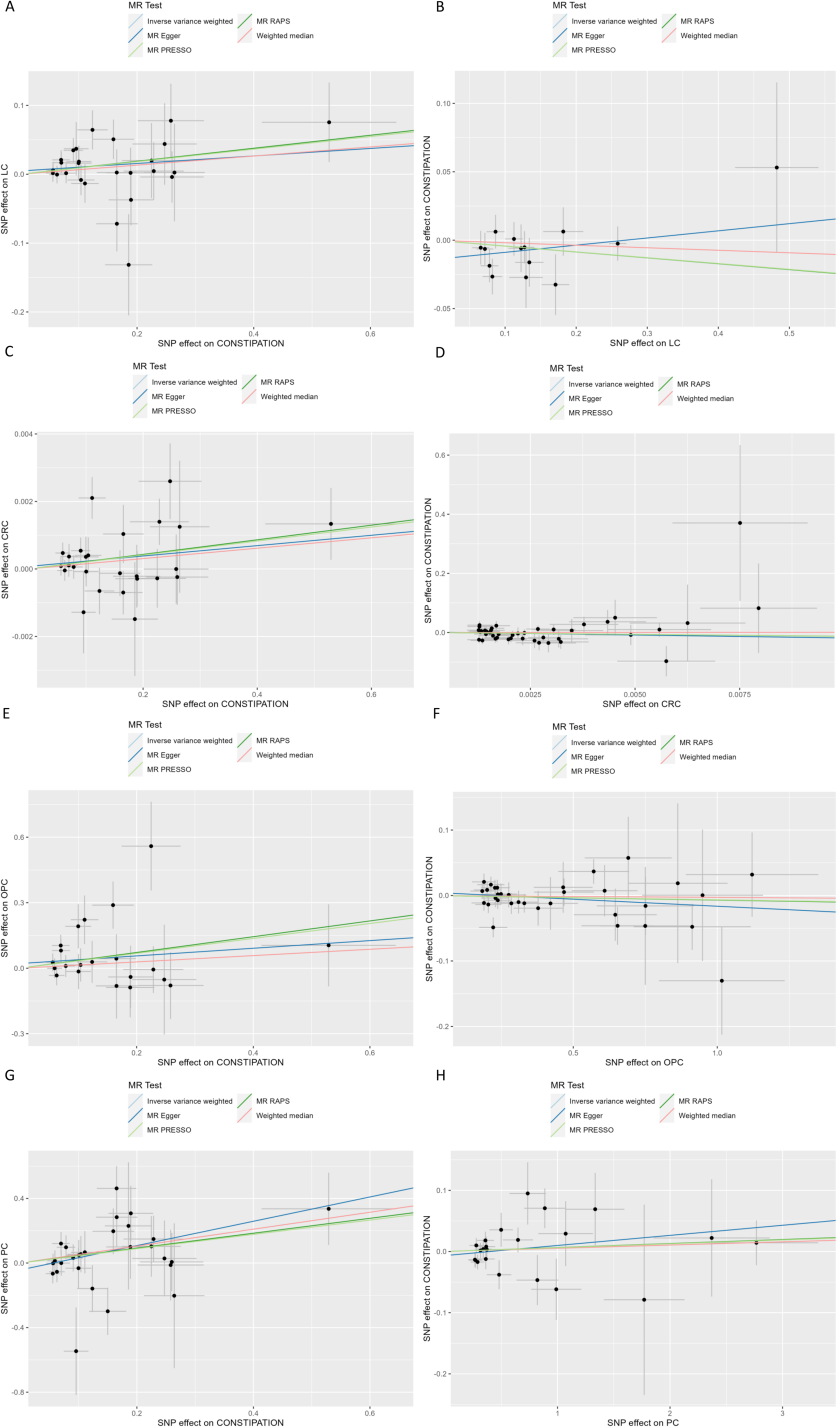
Scatter plot of genetic associations between Constipation and significant Cancer **(A)** Constipation on LC; **(B)** LC on Constipation; **(C)** Constipation on CRC; **(D)** CRC on Constipation; **(E)** Constipation on OPC; **(F)** OPC on Constipation; **(G)** Constipation on PC; **(F)** PC on Constipation; SNP, Single Nucleotide Polymorphism; CRC, Colorectal cancer; LC, Lung cancer; OPC, Oral cavity and pharyngeal cancer; PC, Pancreatic cancer.

Effect of constipation on LC: Taking constipation as the exposure factor and LC as the outcome, 26 independent genome-wide significant SNPs were identified to investigate the causal relationship between constipation and the risk of LC. The results showed IVW (OR=1.0955 (1.0134, 1.1843), P=0.0218), indicating a potential causal relationship between constipation and the risk of LC. Additionally, there was a significant association observed with MR-PRESSO and MR.RAPS (P = 0.0303 and 0.0250, respectively). Although MR-Egger and Weighted Median did not exhibit a significant association, its results align with the direction of the IVW method. (**Table 1**, **Figures 2**, **3**; **Supplementary Table 3**).

**Table 1:** Results of two-sample bidirectional MR analysis of the causal effects between Constipation and Cancer.

Exposures	Outcomes	IVW method					
		Number of SNPs	Beta	SE	OR	95% CI of OR	P-value
Constipation	Lung cancer	26	0.0912	0.0398	1.0955	(1.0134, 1.1843)	0.0218*
	Colorectal cancer	26	0.0021	0.0009	1.0021	(1.0003, 1.0039)	0.0234*
	Oral cavity and pharyngeal cancer	21	0.3413	0.1706	1.4068	(1.0070, 1.9652)	0.0454*
	Pancreatic cancer	28	0.4434	0.1937	1.5580	(1.0659, 2.2773)	0.0221*
	Breast cancer	28	0.0164	0.0251	1.0165	(0.9678, 1.0677)	0.5128
	Skin melanoma	26	-0.0003	0.0008	0.9997	(0.9982, 1.0012)	0.7012
	Bladder cancer	24	0.0000	0.0004	1.0000	(0.9991, 1.0008)	0.9283
	Liver & bile duct cancer	15	0.0003	0.0003	1.0003	(0.9996, 1.0009)	0.3999
	Cervical cancer	20	0.0003	0.0006	1.0003	(0.9991, 1.0015)	0.6090
	Ovarian cancer	24	0.0005	0.0008	1.0005	(0.9990, 1.0021)	0.5046
	Thyroid cancer	21	0.0034	0.0686	1.0034	(0.8771, 1.1479)	0.9604
	Endometrial cancer	27	0.0496	0.0583	1.0509	(0.9373, 1.1782)	0.3950
	Gastric cancer	28	0.4649	0.3840	1.5918	(0.7499, 3.3789)	0.2260
	Esophageal cancer	22	0.0001	0.0003	1.0001	(0.9994, 1.0007)	0.8555
	Prostate cancer	28	-0.0148	0.0307	0.9853	(0.9278, 1.0464)	0.6302
	Brain cancer	20	0.0000	0.0004	1.0000	(0.9993, 1.0007)	0.9612
Lung cancer	Constipation	14	-0.0426	0.0290	0.9583	(0.9054, 1.0143)	0.1417
Colorectal cancer		45	-1.2145	1.2356	0.2968	(0.0263, 3.3446)	0.3257
Oral cavity and pharyngeal cancer		33	-0.0066	0.0107	0.9934	(0.9729, 1.0144)	0.5361
Pancreatic cancer		22	0.0063	0.0086	1.0063	(0.9894, 1.0235)	0.4646
Breast cancer		168	-0.0232	0.0161	0.9771	(0.9468, 1.0084)	0.1494
Skin melanoma		51	0.3739	1.5148	1.4534	(0.0746, 28.3000)	0.8050
Bladder cancer		38	1.9602	2.9303	7.1010	(0.0228, 2216.4089)	0.5035
Liver & bile duct cancer		26	0.0359	6.8071	1.0366	(0.0000, 645492.2107)	0.9958
Cervical cancer		20	-6.1336	4.0456	0.0022	(0.0000, 6.0236)	0.1295
Ovarian cancer		30	1.0850	2.0136	2.9593	(0.0572, 153.1828)	0.5900
Thyroid cancer		19	-0.0267	0.0152	0.9737	(0.9451, 1.0030)	0.0783
Endometrial cancer		65	0.0325	0.0202	1.0330	(0.9930, 1.0747)	0.1067
Gastric cancer		19	-0.0016	0.0054	0.9984	(0.9878, 1.0090)	0.7606
Esophageal cancer		25	1.7268	4.7540	5.6228	(0.0005, 62609.6759)	0.7164
Prostate cancer		170	0.0078	0.0136	1.0078	(0.9813, 1.0350)	0.5668
Brain cancer		36	0.4950	4.7305	1.6405	(0.0002, 17443.9962)	0.9167

MR, Mendelian Randomization; SNP, Single Nucleotide Polymorphism; OR, Odds Ratio; 95%CI, 95% Confidence Interval; IVW, Inverse Variance Weighted.

“*” indicates P < 0.05. Bold values indicate P values that are statistically significant.

Effect of LC on constipation: Considering LC as the exposure factor and constipation as the outcome, 14 independent genome-wide significant SNPs were identified. The results showed IVW (OR=0.9583 (0.9054, 1.0143), P=0.1417), indicating no significant association between LC and the risk of constipation. In addition, none of the other 4 methods yielded evidence of a causal association between LC and the risk of developing constipation (**Table 1**, **Figures 2**, **3**; **Supplementary Table 3**).

### Constipation and CRC

Effect of constipation on CRC: Taking constipation as the exposure factor and CRC as the outcome, 26 independent genome-wide significant SNPs were identified to investigate the causal relationship between constipation and CRC. The results showed evidence of a potential causal relationship between constipation and the risk of CRC using the IVW method (OR=1.0021 (1.0003,1.0039), P= 0.0234). Additionally, there was a significant association observed with MR-PRESSO and MR.RAPS (P = 0.0323 and 0.0153, respectively). Although MR-Egger and Weighted Median did not exhibit a significant association, its results align with the direction of the IVW method. (**Table 1**, **Figures 2**, **3**; **Supplementary Table 3**).

Effect of CRC on constipation: Considering CRC as the exposure factor and constipation as the outcome, 45 independent genome-wide significant SNPs were identified. The results from the IVW method (OR=0.2968 (0.0263,3.3446), P= 0.3257) showed no significant association between CRC and the risk of constipation. Additionally, the other four methods did not provide evidence of a causal relationship between CRC and the risk of constipation (**Table 1**, **Figures 2**, **3**; **Supplementary Table 3**).

### Constipation and OPC

Effect of constipation on OPC: Taking constipation as the exposure factor and OPC as the outcome, 21 independent genome-wide significant SNPs were identified to investigate the causal relationship between constipation and the risk of OPC. The results showed IVW (OR=1.4068 (1.0070, 1.9652), P=0.0454), indicating a potential causal relationship between constipation and the risk of OPC. Additionally, there was a significant association observed with MR.RAPS (P = 0.0219, respectively). Although the other 3 methods did not exhibit a significant association, its results align with the direction of the IVW method (**Table 1**, **Figures 2**, **3**; **Supplementary Table 3**).

Effect of OPC on constipation: Considering OPC as the exposure factor and constipation as the outcome, 33 independent genome-wide significant SNPs were identified. The results showed IVW (OR=0.9934 (0.9729, 1.0144), P=0.5361), demonstrating that there was no significant association between OPC and the risk of constipation. In addition, none of the other 4 methods yielded evidence of a causal association between OPC and the risk of developing constipation (**Table 1**, **Figures 2**, **3**; **Supplementary Table 3**).

### Constipation and PC

Effect of constipation on PC: Taking constipation as the exposure factor and PC as the outcome, 28 independent genome-wide significant SNPs were identified to investigate the causal relationship between constipation and the risk of PC. The results showed IVW (OR=1.5580 (1.0659,2.2773), P=0.0221), indicating a potential causal relationship between constipation and the risk of PC. Additionally, there was a significant association observed with MR Egger, Weighted median, MR-PRESSO and MR.RAPS (P = 0.0407,0.0392,0.0301 and 0.0115, respectively), its results align with the direction of the IVW method (**Table 1**, **Figures 2**, **3**; **Supplementary Table 3**).

Effect of PC on constipation: Considering PC as the exposure factor and constipation as the outcome, 22 independent genome-wide significant SNPs were identified. The results showed IVW (OR=1.0063 (0.9894,1.0235), P=0.4646), indicating that there was no significant association between PC and the risk of constipation. In addition, none of the other 4 methods yielded evidence of a causal association between PC and the risk of developing constipation (**Table 1**, **Figures 2**, **3**; **Supplementary Table 3**).

### Constipation and other cancers

In addition, we also analyzed the bidirectional causal relationship between constipation and 12 other types of cancers, including gastric cancer, esophageal cancer, skin melanoma, thyroid cancer, breast cancer, liver & bile duct cancer, endometrial cancer, bladder cancer, cervical cancer, brain cancer, ovarian cancer and prostate cancer. However, there was no evidence to support a causal relationship between them in all five methods. For specific analysis results, please refer to **Supplementary Table 3**. The results of the forest plot and scatter plot can be found in **Supplementary Figure 1** and **Supplementary Figure 2**.

### sensitivity analysis

Cochran’s Q test evaluated heterogeneity among instrumental variable estimates for individual genetic variants. The results indicated no evidence of heterogeneity (Q > 0.05) except for the analysis on the effect of prostate cancer and gastric cancer on the risk of constipation. A random-effects IVW method was used to estimate the causal association when heterogeneity exists. The intercept p-value obtained from the MR Egger regression and global test p-value of MR-PRESSO indicated no potential pleiotropy of IVs (p > 0.05) except for the analysis on the effect of gastric cancer and prostate cancer on the risk of constipation. MR-PRESSO suggested that there was significant horizontal pleiotropy between the instrumental variables of gastric cancer and outcome (P = 0.009), and rs72813957 was identified as outlier. However, the results did not change significantly after removing the SNP (OR = 0.9947 (0.9853, 1.0042), P = 0.2914). In the analysis of the effect of prostate cancer on the risk of constipation, MR-PRESSO found there was significant horizontal pleiotropy (P = 0.002) and rs1048169 was identified as a pleiotropic SNP. After removing the outlier, the results did not change substantially (OR = 1.0122 (0.9860, 1.0392), P = 0.3657). For detailed information on heterogeneity and pleiotropy analyses, please refer to **Supplementary Table 4**. Funnel plots (**Supplementary Figure 3**) showed no significant asymmetry in causal effects when using single SNPs as IVs, suggesting that the results were unlikely to be affected by potential bias. Leave-one-out analysis indicated that removing each SNP to assess its effect on IVW point estimates did not significantly affect the overall results (**Supplementary Figure 4**).

## Discussion

This study used a bidirectional two-sample MR method to investigate the association between constipation and 16 common types of cancers in the European population. The forward MR results showed that constipation was associated with an increased risk of LC, CRC, OPC and PC, suggesting that constipation may be a potential risk factor for these cancers. Sensitivity tests showed that the causal effects were not caused by outliers or horizontal pleiotropy. However, although the IVW method suggests that constipation may be associated with an increased risk of LC, CRC and OPC, the results from the Weighted Median and MR-Egger methods did not reach statistical significance, indicating a possible violation of the fundamental assumptions. Therefore, these results should be considered preliminary findings that require further validation in future studies. Additionally, no association was observed between constipation and the risk of gastric cancer, esophageal cancer, skin melanoma, thyroid cancer, breast cancer, liver & bile duct cancer, endometrial cancer, bladder cancer, cervical cancer, brain cancer, ovarian cancer and prostate cancer. Furthermore, the reverse MR results did not provide any evidence supporting a causal effect of cancer on the risk of constipation. To our knowledge, this is the first study focusing on the causal association between constipation and pan-cancer risk using MR analysis.

Our finding showed potential association between constipation and the risk of CRC, which was consistent with a retrospective study in the United States, which found a significantly higher incidence of CRC in patients with chronic constipation compared to matched individuals without constipation (16). In addition, our study indicates a potential effect of constipation on the risk of OPC,LC and PC. However, the mechanisms behind the above associations remain unclear. Chronic constipation may lead to prolonged exposure of the colon to potential carcinogens, such as bile acids, which can increase the risk of CRC (12). Imbalance in the gut microbiota caused by constipation may also be one possible explanation for the increased risk of cancer. The imbalance of microbiota could disrupt the intestinal mucosal barrier, trigger inflammation, cytokine release, and immune suppression, and affect the host’s physiological activity through the metabolites of gut microbiota. Elevated levels of inflammation may further exacerbate the disruption of gut microbiota, leading to abnormal intestinal and chronic diseases, including cancer (17).

Studies have shown that compared to healthy individuals, patients with constipation have significantly lower abundance of *Bifidobacterium*, *Lactobacillus*, and butyrate-producing bacteria, while the abundance of *E. coli* increases (31, 32). *E. coli* may be involved in in the occurrence and progression of CRC by inducing abnormal expression of proto-oncogenes and oncogenes, as well as abnormal mismatches in chromosomal repair (33). Lower levels of butyrate-producing bacteria may also contribute to the progression of CRC (34). *Bifidobacteria* have a protective effect against inflammation induced by TNF-α and lipopolysaccharide (LPS), while TNF-α can promote LC metastasis by inducing epithelial mesenchymal transition (35, 36). In addition, as an important product of butyrate-producing bacteria, butyrate has been associated with anti-inflammatory activity, cell proliferation, induction of regulatory T cell differentiation and apoptosis (37, 38). Butyrate can enhance ferroptosis induced by erastin in LC cells by upregulating ATF3 expression to reduce the expression level of SLC7A11, thereby inhibiting the growth of lung cancer cells (39). Therefore, we hypothesized that the lower levels of *Bifidobacterium* and butyrate-producing bacteria in constipated patients may be associated with the progression of LC. *Lactobacilli* exhibit inhibitory effects on the occurrence and progression of cancer, and a lower abundance of *Lactobacillus* may promote the progression of OPC (40). Previous studies have shown that intervention with *Clostridium butyricum* or its metabolite butyrate triggered superoxidative stress and intracellular lipid accumulation, which was linked to a better prognosis and less aggressive features of Pancreatic ductal adenocarcinoma (41). *Lactobacillus casei* combined with *Lactobacillus reuteri* alleviate PC by inhibiting TLR4 to promote macrophage M1 polarization and regulate gut microbial homeostasis (42). TUBB (tubulin, beta class I) may be associated with the pathogenic *E. coli* infection, which may be involved in the carcinogenesis and progression of PC by activating the TUBB/Rho/ROCK signaling pathway (43). Therefore, the lower levels of *Lactobacillus* and butyrate-producing bacteria, along with increased abundance of *E. coli* in patients with constipation, may contribute to the progression of PC. Alterations and imbalances in the composition of the microbiome are considered to have genetic toxic potential, produce various gene toxins, promote the generation of radicals, which can affect DNA repair, causing DNA damage, cell cycle arrest and apoptosis, and initiating carcinogenesis in organisms (44). Therefore, we hypothesize that alterations in the microbiome caused by constipation may drive the progression of CRC, LC,OPC and PC.

On the other hand, aquaporin 3 (AQP3), transforming growth factor-beta (TGF-β) and related signaling pathways may contribute to the pathogenesis of constipation (45, 46). Studies have shown that AQP3 is overexpressed in LC, CRC, oral squamous cell carcinomas and pancreatic ductal adenocarcinoma (47). AQP3 may affect tumor progression by reducing differentiation and inhibiting apoptosis of LC stem cells through the Wnt/GSK-3β/β-catenin pathway (48). In human CRC cells, overexpression of AQP3 can promotes cell migration, indicating tumor metastasis and poor prognosis in CRC (49). AQP3 can promote tumor growth of pancreatic cancer cells by activating the Mtor signaling pathway (50). Disruption of TGF-β signaling usually promotes tumor formation at an early stage, while its activation may promote invasion and metastasis in CRC. Furthermore, its activation in the tumor microenvironment typically suppresses tumor immunity and supports cancer cell survival (51). Constipation is also a clinical symptom of some common gastrointestinal disorders such as irritable bowel syndrome (IBS) and inflammatory bowel disease (IBD).Patients with IBD have a significantly increased risk of CRC, mainly caused by the pro-tumorigenic effects of chronic intestinal inflammation (52). The expression and activity of G Protein-Coupled Estrogen Receptor (GPER) is associated with intestinal motility as well as the development and progression of intestinal diseases, including IBD, IBS, and CRC (53). These findings may provide insights into the potential mechanisms between constipation and the risks of CRC, LC, OPC and PC. However, the causal association between constipation and cancer needs to be interpreted with caution. Future research is necessary to further investigate its underlying mechanisms.

It is noteworthy that previous observational study has implicated constipation as a potential risk factor for breast cancer (17). Specifically, a study utilizing data from 11,217 individuals in the National Health and Nutrition Examination Survey identified an association between constipation and an increased risk of breast cancer. However, our study did not provide any evidence supporting a causal association between constipation and the risk of breast cancer. This contradicts the findings of existing research. It is hypothesized that there may be coincidental associations or confounding influenced by some undetermined factors.

Our study has several significant strengths. To our knowledge, this is the first study of the genetic effect of constipation on the risk of pan-cancer. In addition, we used a bidirectional MR method to assess the independent causal effect of constipation on cancer, mitigating the influence of reverse causation or residual confounding factors. Finally, we used GWAS with large sample sizes whenever possible to ensure the robustness and reliability of our MR analyses.

Inevitably, our study has some limitations. Firstly, the GWAS data used in our study were derived from participants of European ancestry, potentially limiting the generalizability of our findings to other populations. Therefore, these findings should be further validated in more diverse populations. Secondly, despite meticulous selection of genetic variants associated with constipation and cancer from GWAS, completely excluding pleiotropy presents challenges. Given the absence of horizontal pleiotropy in our study, the causal effect estimates are considered robust. Thirdly, due to the lack of GWAS data on different types of constipation, our study is limited to exploring the association between constipation (K59.0) and cancer. Therefore, the future researches are required to investigate the association between different types of constipation and cancer on a more detailed data basis. Fourthly, the “control” group in the cancer GWAS datasets includes patients with other types of cancer. However, due to limitations in the datasets, we were unable to exclude patients with other cancers from all “control” groups. Consequently, this confounding factor may introduce some bias. Furthermore, the potential impact of different adjustments in the original GWAS on MR analysis, we need a cautious interpretation of our findings. Finally, MR methods can only analyze causal association and do not explain the mechanisms behind the increased risk of certain cancers associated with constipation. Further experimental studies are required to explore the mechanisms of the effect of constipation on cancer risk.

## Conclusions

In conclusion, our bidirectional MR study suggests a potential association between constipation and increased risk of CRC, LC, OPC and PC, providing crucial evidence for clinicians in formulating effective prevention strategies. Nonetheless, our study still has limitations that are worth considering when interpreting the findings. Further investigation is necessary to validate our results and to investigate the underlying biological mechanisms.

## Data Availability

The original contributions presented in the study are included in the article/**Supplementary Material**. Further inquiries can be directed to the corresponding author/s.
