# Supplementary material for: Causal associations between constipation and pan-cancer: a bidirectional Mendelian randomization study
Source: Front Oncol. 2024 Sep 13;14:1428003. doi: 10.3389/fonc.2024.1428003 (PMC11427234; doi:10.3389/fonc.2024.1428003)
Supplement: Supplementary file 1 [file DataSheet1.pdf]

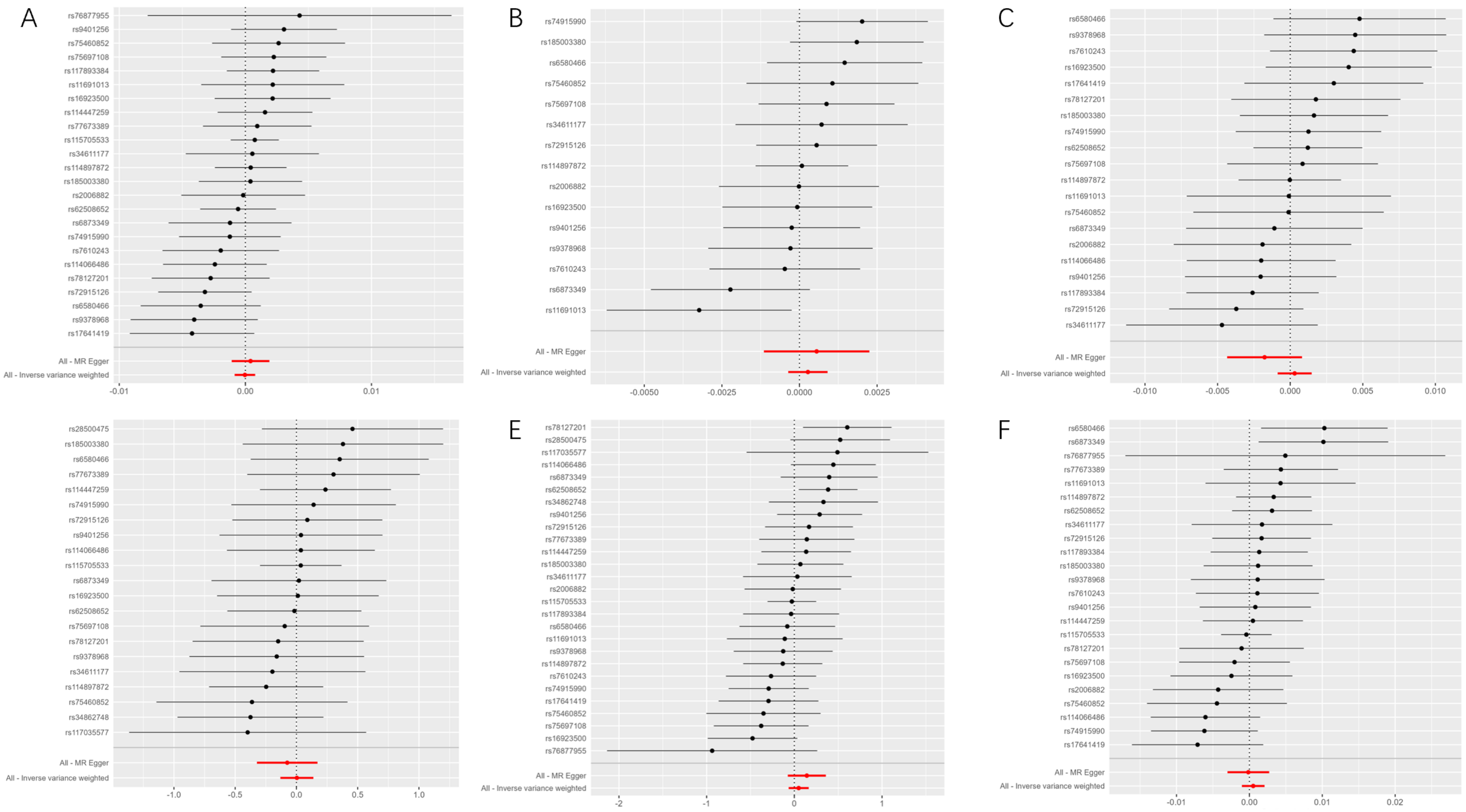
A
rs76877955
rs9401256
rs75460852
rs75697108
rs117893384
rs11691013
rs16923500
rs114447259
rs77673389
rs115705533
rs34611177
rs114897872
rs185003380
rs2006882
rs62508652
rs6873349
rs74915990
rs7610243
rs114066486
rs78127201
rs72915126
rs6580466
rs9378968
rs17641419
All - MR Egger
All - Inverse variance weighted
-0.01
0.00
0.01
B
rs74915990
rs185003380
rs6580466
rs75460852
rs75697108
rs34611177
rs72915126
rs114897872
rs2006882
rs16923500
rs9401256
rs9378968
rs7610243
rs6873349
rs11691013
All - MR Egger
All - Inverse variance weighted
-0.0050
-0.0025
0.0000
0.0025
C
rs6580466
rs9378968
rs7610243
rs16923500
rs17641419
rs78127201
rs185003380
rs74915990
rs62508652
rs75697108
rs114897872
rs11691013
rs75460852
rs6873349
rs2006882
rs114066486
rs9401256
rs117893384
rs72915126
rs34611177
All - MR Egger
All - Inverse variance weighted
-0.010
-0.005
0.000
0.005
0.010
rs28500475
rs185003380
rs6580466
rs77673389
rs114447259
rs74915990
rs72915126
rs9401256
rs114066486
rs115705533
rs6873349
rs16923500
rs62508652
rs75697108
rs78127201
rs9378968
rs34611177
rs114897872
rs75460852
rs34862748
rs117035577
All - MR Egger
All - Inverse variance weighted
-1.0
-0.5
0.0
0.5
1.0
E
rs78127201
rs28500475
rs117035577
rs114066486
rs6873349
rs62508652
rs34862748
rs9401256
rs72915126
rs77673389
rs114447259
rs185003380
rs34611177
rs2006882
rs115705533
rs117893384
rs6580466
rs11691013
rs9378968
rs114897872
rs7610243
rs74915990
rs17641419
rs75460852
rs75697108
rs16923500
rs76877955
All - MR Egger
All - Inverse variance weighted
-2
-1
0
1
F
rs6580466
rs6873349
rs76877955
rs77673389
rs11691013
rs114897872
rs62508652
rs34611177
rs72915126
rs117893384
rs185003380
rs9378968
rs7610243
rs9401256
rs114447259
rs115705533
rs78127201
rs75697108
rs16923500
rs2006882
rs75460852
rs114066486
rs74915990
rs17641419
All - MR Egger
All - Inverse variance weighted
-0.01
0.00
0.01
0.02

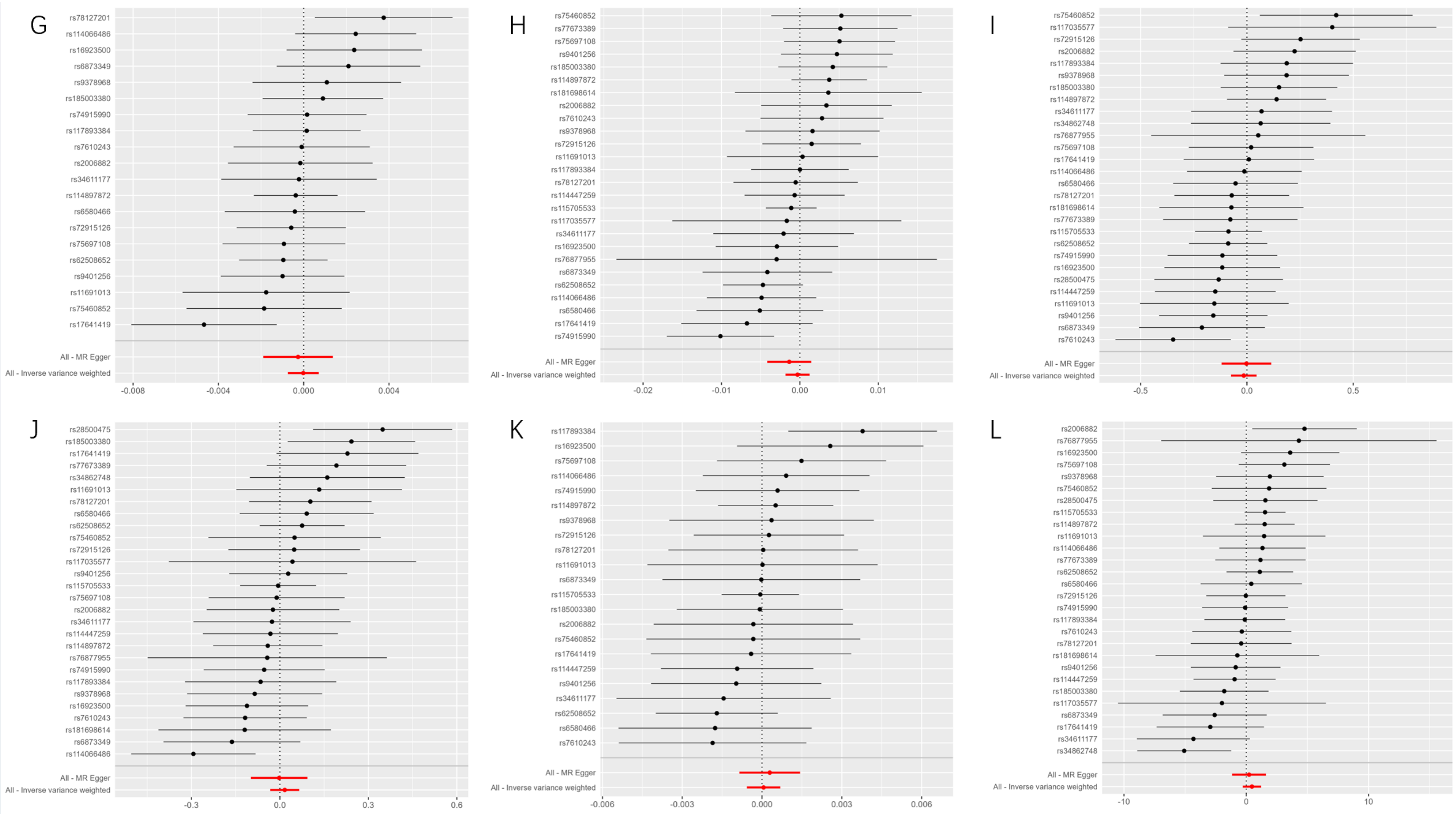
G
rs78127201
rs114066486
rs16923500
rs6873349
rs9378968
rs185003380
rs74915990
rs117893384
rs7610243
rs2006882
rs34611177
rs114897872
rs6580466
rs72915126
rs75697108
rs62508652
rs9401256
rs11691013
rs75460852
rs17641419
All - MR Egger
All - Inverse variance weighted
-0.008
-0.004
0.000
0.004
H
rs75460852
rs77673389
rs75697108
rs9401256
rs185003380
rs114897872
rs181698614
rs2006882
rs7610243
rs9378968
rs72915126
rs11691013
rs117893384
rs78127201
rs114447259
rs115705533
rs117035577
rs34611177
rs16923500
rs76877955
rs6873349
rs62508652
rs114066486
rs6580466
rs17641419
rs74915990
All - MR Egger
All - Inverse variance weighted
-0.02
-0.01
0.00
0.01
I
rs75460852
rs117035577
rs72915126
rs2006882
rs117893384
rs9378968
rs185003380
rs114897872
rs34611177
rs34862748
rs76877955
rs75697108
rs17641419
rs114066486
rs6580466
rs78127201
rs181698614
rs77673389
rs115705533
rs62508652
rs74915990
rs16923500
rs28500475
rs114447259
rs11691013
rs9401256
rs6873349
rs7610243
All - MR Egger
All - Inverse variance weighted
-0.5
0.0
0.5
J
rs28500475
rs185003380
rs17641419
rs77673389
rs34862748
rs11691013
rs78127201
rs6580466
rs62508652
rs75460852
rs72915126
rs117035577
rs9401256
rs115705533
rs75697108
rs2006882
rs34611177
rs114447259
rs114897872
rs76877955
rs74915990
rs117893384
rs9378968
rs16923500
rs7610243
rs181698614
rs6873349
rs114066486
All - MR Egger
All - Inverse variance weighted
-0.3
0.0
0.3
0.6
K
rs117893384
rs16923500
rs75697108
rs114066486
rs74915990
rs114897872
rs9378968
rs72915126
rs78127201
rs11691013
rs6873349
rs115705533
rs185003380
rs2006882
rs75460852
rs17641419
rs114447259
rs9401256
rs34611177
rs62508652
rs6580466
rs7610243
All - MR Egger
All - Inverse variance weighted
-0.006
-0.003
0.000
0.003
0.006
L
rs2006882
rs76877955
rs16923500
rs75697108
rs9378968
rs75460852
rs28500475
rs115705533
rs114897872
rs11691013
rs114066486
rs77673389
rs62508652
rs6580466
rs72915126
rs74915990
rs117893384
rs7610243
rs78127201
rs181698614
rs9401256
rs114447259
rs185003380
rs117035577
rs6873349
rs17641419
rs34611177
rs34862748
All - MR Egger
All - Inverse variance weighted
-10
0
10

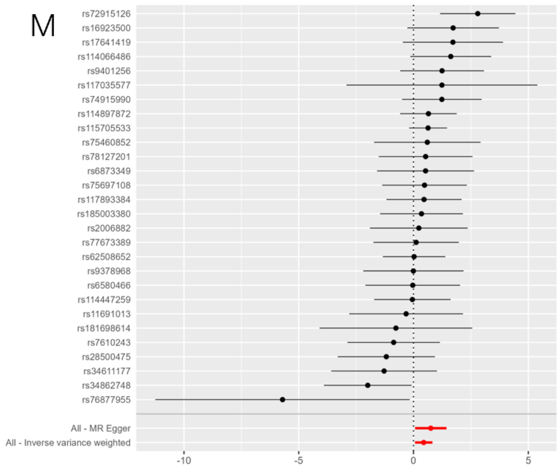


**Supplementary Figure 1-1. Forest plot of MR effect of Constipation on (A) Bladder cancer; (B) Liver & bile duct cancer; (C) Cervical cancer; (D) Thyroid cancer; (E) Endometrial cancer; (F) Ovarian cancer(G) Brain cancer; (H) Skin melanoma; (I) Prostate cancer; (J) Breast cancer; (K) Esophageal cancer; (L) Gastric cancer; (M) Pancreatic cancer;**

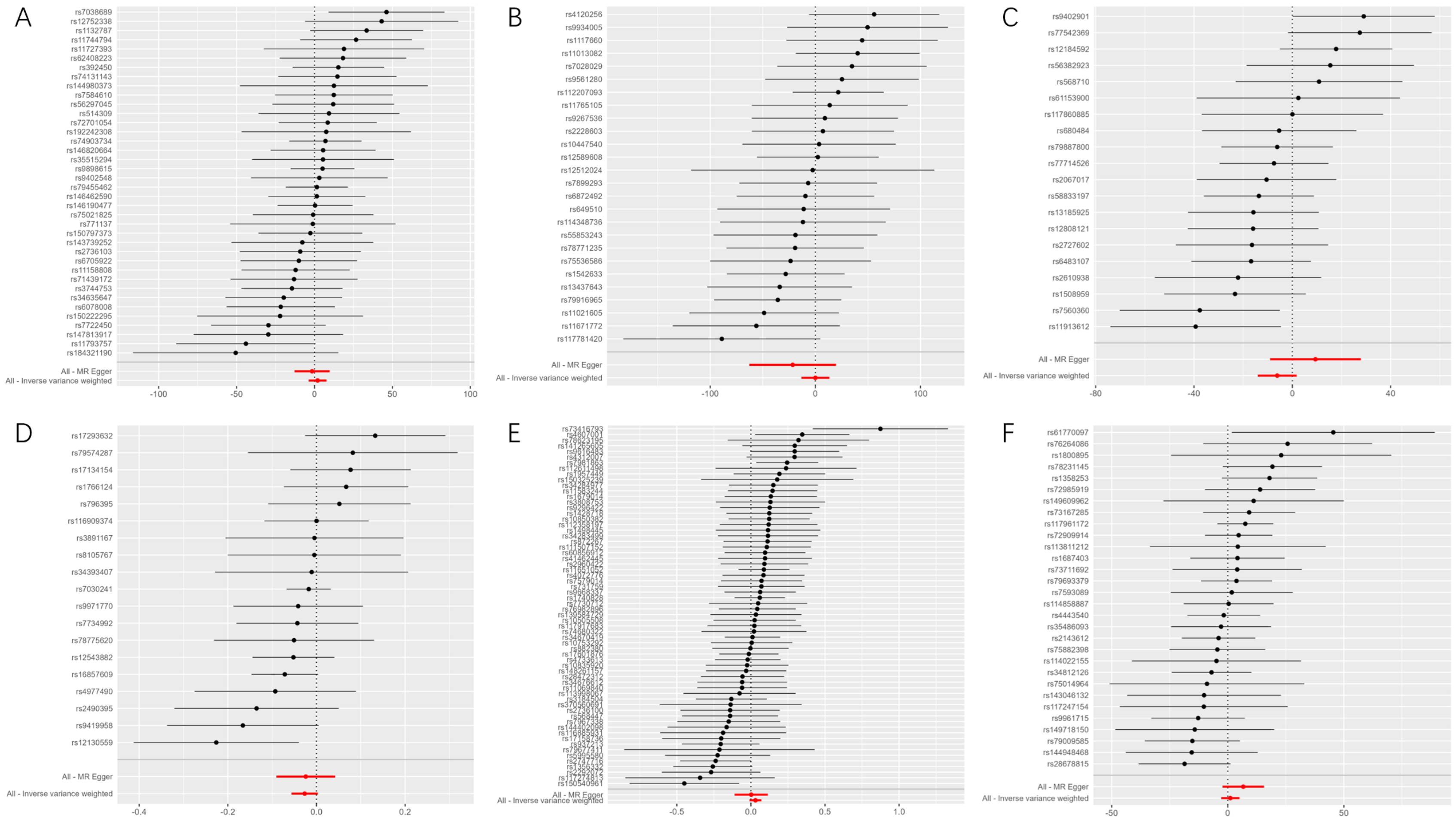
A
rs7038689
rs12752338
rs1132787
rs11744794
rs11727393
rs62408223
rs392450
rs74131143
rs144980373
rs7584610
rs56297045
rs514309
rs72701054
rs192242308
rs74903734
rs146820664
rs35515294
rs9898615
rs9402548
rs79455462
rs146462590
rs146190477
rs75021825
rs771137
rs150797373
rs143739252
rs2736103
rs6705922
rs11158808
rs71439172
rs3744753
rs34635647
rs6078008
rs150222295
rs7722450
rs147813917
rs11793757
rs184321190
All - MR Egger
All - Inverse variance weighted
-100
-50
0
50
100
B
rs4120256
rs9934005
rs1117660
rs11013082
rs7028029
rs9561280
rs112207093
rs11765105
rs9267536
rs2228603
rs10447540
rs12589608
rs12512024
rs7899293
rs6872492
rs649510
rs114348736
rs55853243
rs78771235
rs75536586
rs1542633
rs13437643
rs79916965
rs11021605
rs11671772
rs117781420
All - MR Egger
All - Inverse variance weighted
-100
0
100
C
rs9402901
rs77542369
rs12184592
rs56382923
rs568710
rs61153900
rs117860885
rs680484
rs79887800
rs77714526
rs2067010
rs58833197
rs13185925
rs12808121
rs2727602
rs6483107
rs2610938
rs1508959
rs7560360
rs11913612
All - MR Egger
All - Inverse variance weighted
-80
-40
0
40
D
rs17293632
rs79574287
rs17134154
rs1766124
rs796395
rs116909374
rs3891167
rs8105767
rs34393407
rs7030241
rs9971770
rs7734992
rs78775620
rs12543882
rs16857609
rs4977490
rs2490395
rs9419958
rs12130559
All - MR Egger
All - Inverse variance weighted
-0.4
-0.2
0.0
0.2
E
rs73416793
All - MR Egger
All - Inverse variance weighted
-0.5
0.0
0.5
1.0
F
rs61770097
rs76264086
rs1800895
rs78231145
rs1358253
rs72985919
rs149609962
rs73167285
rs117961172
rs72909914
rs113811212
rs1687403
rs73711692
rs79693379
rs7593089
rs114858887
rs4443540
rs35486093
rs2143612
rs75882398
rs114022155
rs34812126
rs75014964
rs143046132
rs117247154
rs9961715
rs149718150
rs79009585
rs144948468
rs28678815
All - MR Egger
All - Inverse variance weighted
-50
0
50

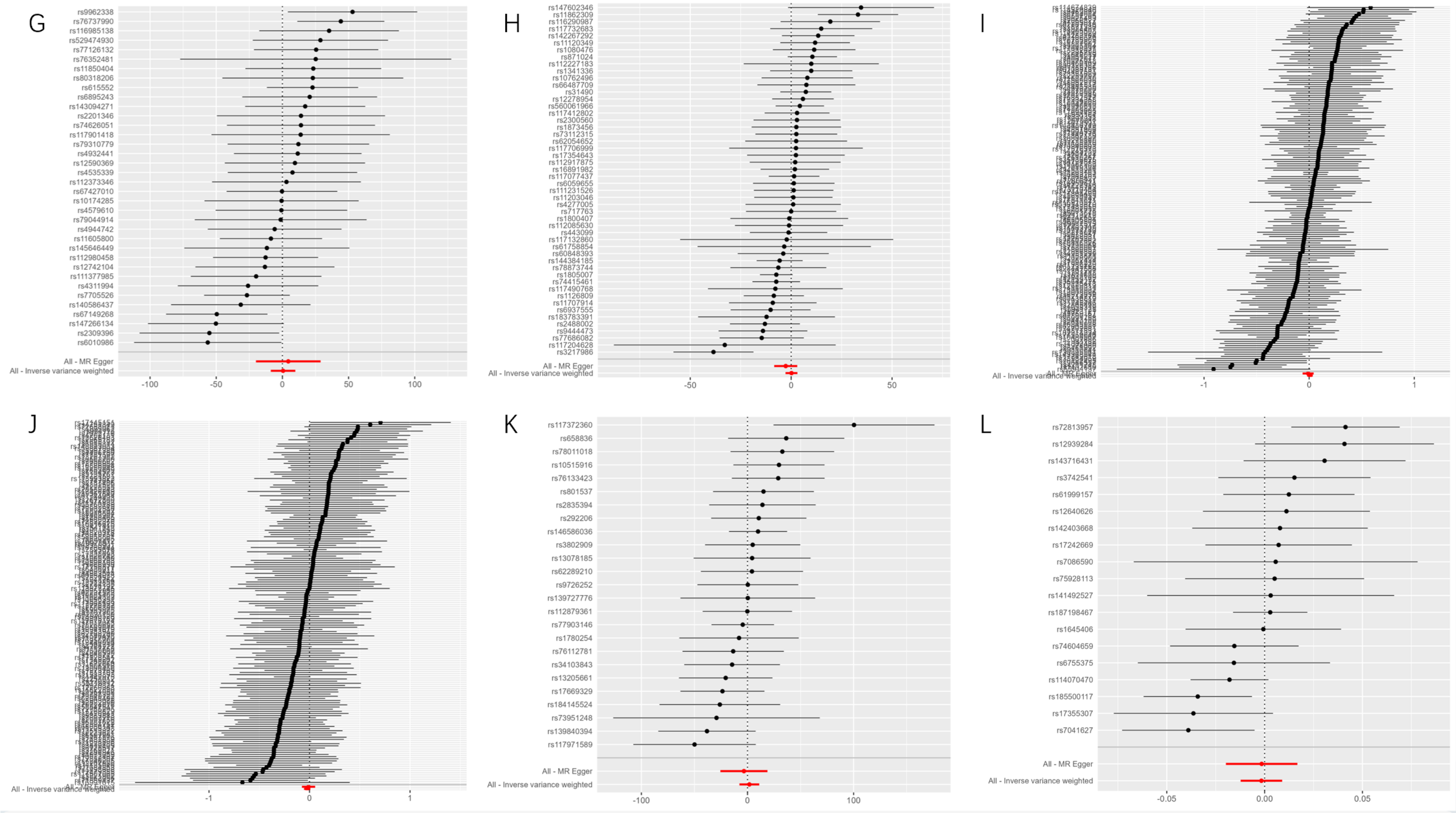
G
rs9962338
rs76737990
rs116985138
rs529474930
rs77126132
rs76352481
rs11850404
rs80318206
rs615552
rs6895243
rs143094271
rs2201346
rs74626051
rs117901418
rs79310779
rs4932441
rs12590369
rs4535339
rs112373346
rs67427010
rs10174285
rs4579610
rs79044914
rs4944742
rs11605800
rs145646449
rs112980458
rs12742104
rs111377985
rs4311994
rs7705526
rs140586437
rs67149268
rs147266134
rs2309396
rs6010986
All - MR Egger
All - Inverse variance weighted
-100
-50
0
50
100
H
rs147602346
rs11862309
rs116290987
rs117732683
rs142267292
rs11120349
rs1080476
rs871024
rs112227183
rs1341336
rs10762496
rs66487709
rs31490
rs12278954
rs560061966
rs117412802
rs2300560
rs1873456
rs73112315
rs62054652
rs117706999
rs17354643
rs112917875
rs16891982
rs117077437
rs6059655
rs111231526
rs11203046
rs4277005
rs717763
rs1800407
rs112085630
rs443099
rs117132860
rs61758854
rs60848393
rs144384185
rs78873744
rs1805007
rs74415461
rs117490768
rs1126809
rs11707914
rs6937555
rs183783391
rs2488002
rs9444473
rs77686082
rs117204628
rs3217986
All - MR Egger
All - Inverse variance weighted
-50
0
50
I
All - Inverse variance weighted
-1
0
1
J
All - Inverse variance weighted
-1
0
1
K
rs117372360
rs658836
rs78011018
rs10515916
rs76133423
rs801537
rs2835394
rs292206
rs146586036
rs3802909
rs13078185
rs62289210
rs9726252
rs139727776
rs112879361
rs77903146
rs1780254
rs76112781
rs34103843
rs13205661
rs17669329
rs184145524
rs73951248
rs139840394
rs117971589
All - MR Egger
All - Inverse variance weighted
-100
0
100
L
rs72813957
rs12939284
rs143716431
rs3742541
rs61999157
rs12640626
rs142403668
rs17242669
rs7086590
rs75928113
rs141492527
rs187198467
rs1645406
rs74604659
rs6755375
rs114070470
rs185500117
rs17355307
rs7041627
All - MR Egger
All - Inverse variance weighted
-0.05
0.00
0.05

M

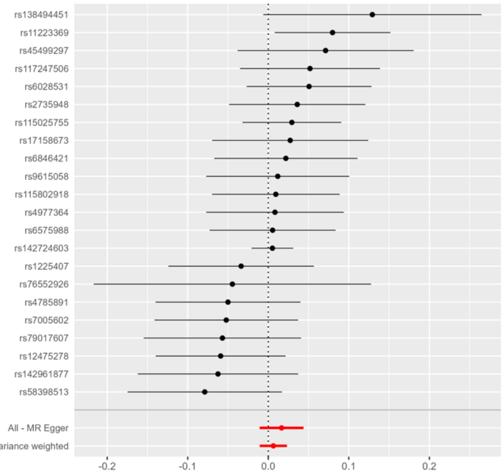


**Supplementary Figure 1-2. Forest plot of MR effect of (A) Bladder cancer; (B) Liver & bile duct cancer; (C) Cervical cancer; (D) Thyroid cancer; (E) Endometrial cancer; (F) Ovarian cancer(G) Brain cancer; (H) Skin melanoma; (I) Prostate cancer; (J) Breast cancer; (K) Esophageal cancer; (L) Gastric cancer; (M) Pancreatic cancer on Constipation**

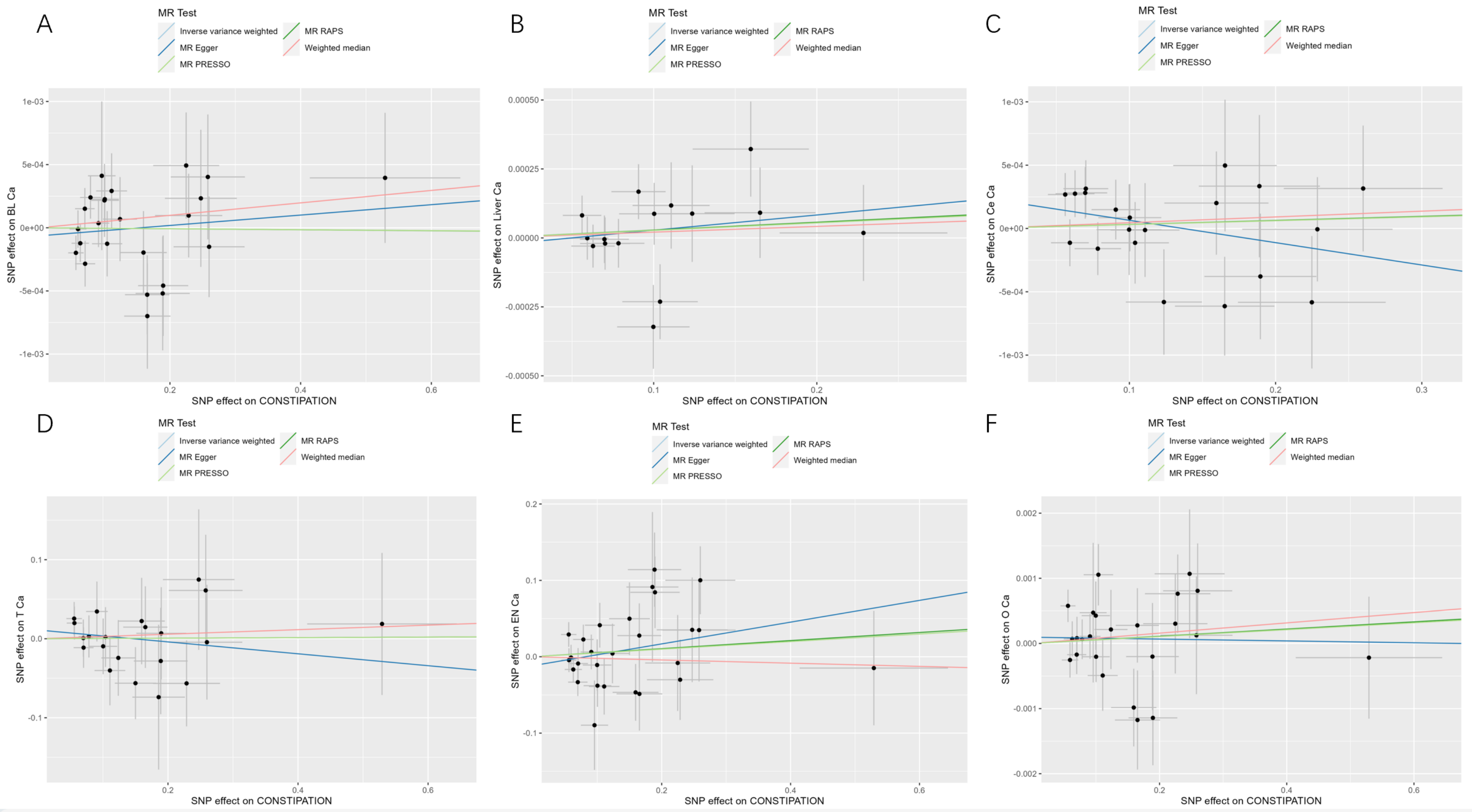
A
MR Test
Inverse variance weighted
MR Egger
MR PRESSO
MR RAPS
Weighted median
SNP effect on BL Ca
SNP effect on CONSTIPATION
1e-03
5e-04
0e+00
-5e-04
-1e-03
0.2
0.4
0.6
B
MR Test
Inverse variance weighted
MR Egger
MR PRESSO
MR RAPS
Weighted median
SNP effect on Liver Ca
SNP effect on CONSTIPATION
0.00050
0.00025
0.00000
-0.00025
-0.00050
0.1
0.2
C
MR Test
Inverse variance weighted
MR Egger
MR PRESSO
MR RAPS
Weighted median
SNP effect on Ce Ca
SNP effect on CONSTIPATION
1e-03
5e-04
0e+00
-5e-04
-1e-03
0.1
0.2
0.3
D
MR Test
Inverse variance weighted
MR Egger
MR PRESSO
MR RAPS
Weighted median
SNP effect on T Ca
SNP effect on CONSTIPATION
0.1
0.0
-0.1
0.2
0.4
0.6
E
MR Test
Inverse variance weighted
MR Egger
MR PRESSO
MR RAPS
Weighted median
SNP effect on EN Ca
SNP effect on CONSTIPATION
0.2
0.1
0.0
-0.1
0.2
0.4
0.6
F
MR Test
Inverse variance weighted
MR Egger
MR PRESSO
MR RAPS
Weighted median
SNP effect on O Ca
SNP effect on CONSTIPATION
0.002
0.001
0.000
-0.001
-0.002
0.2
0.4
0.6

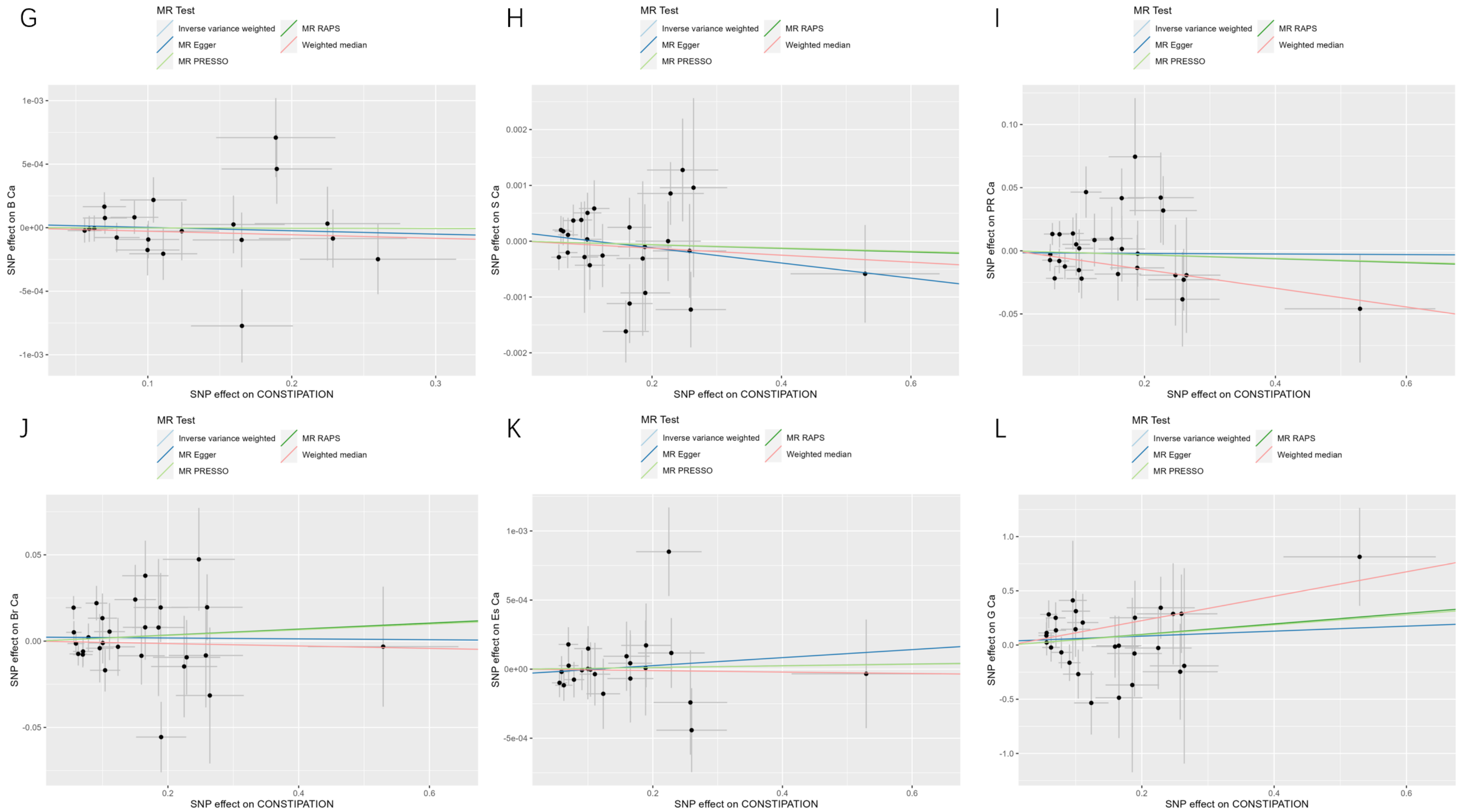
G
MR Test
Inverse variance weighted
MR Egger
MR PRESSO
MR RAPS
Weighted median
SNP effect on B Ca
SNP effect on CONSTIPATION
H
MR Test
Inverse variance weighted
MR Egger
MR PRESSO
MR RAPS
Weighted median
SNP effect on S Ca
SNP effect on CONSTIPATION
I
MR Test
Inverse variance weighted
MR Egger
MR PRESSO
MR RAPS
Weighted median
SNP effect on PR Ca
SNP effect on CONSTIPATION
J
MR Test
Inverse variance weighted
MR Egger
MR PRESSO
MR RAPS
Weighted median
SNP effect on Br Ca
SNP effect on CONSTIPATION
K
MR Test
Inverse variance weighted
MR Egger
MR PRESSO
MR RAPS
Weighted median
SNP effect on Es Ca
SNP effect on CONSTIPATION
L
MR Test
Inverse variance weighted
MR Egger
MR PRESSO
MR RAPS
Weighted median
SNP effect on G Ca
SNP effect on CONSTIPATION

M

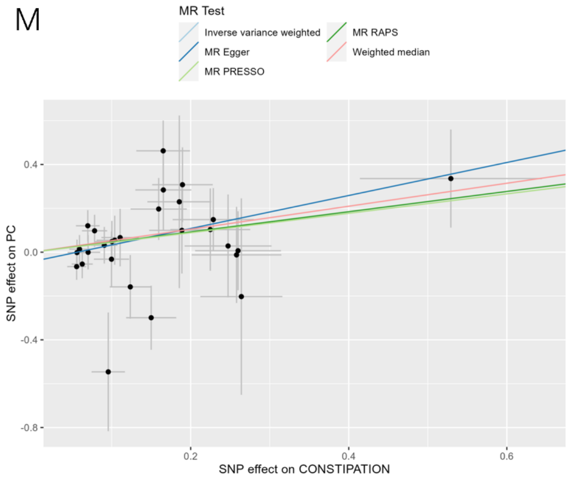


**Supplementary Figure 2-1 | Scatter plot of SNPs associated with Constipation and risk on (A) Bladder cancer; (B) Liver & bile duct cancer; (C) Cervical cancer; (D) Thyroid cancer; (E) Endometrial cancer; (F) Ovarian cancer(G) Brain cancer; (H) Skin melanoma; (I) Prostate cancer; (J) Breast cancer; (K) Esophageal cancer; (L) Gastric cancer; (M) Pancreatic cancer**

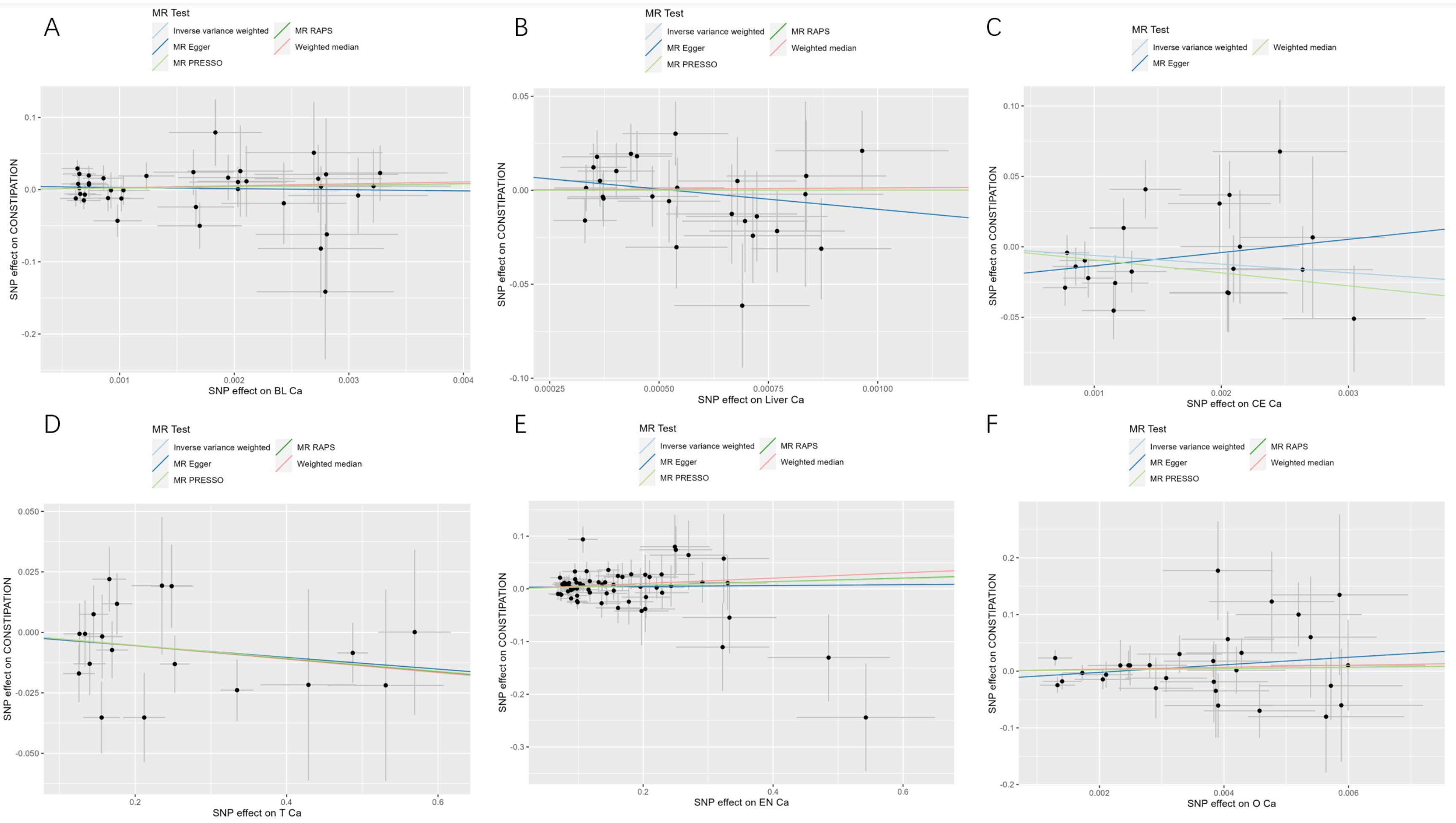
A
MR Test
Inverse variance weighted
MR Egger
MR PRESSO
MR RAPS
Weighted median
SNP effect on CONSTIPATION
SNP effect on BL Ca
B
MR Test
Inverse variance weighted
MR Egger
MR PRESSO
MR RAPS
Weighted median
SNP effect on CONSTIPATION
SNP effect on Liver Ca
C
MR Test
Inverse variance weighted
MR Egger
Weighted median
SNP effect on CONSTIPATION
SNP effect on CE Ca
D
MR Test
Inverse variance weighted
MR Egger
MR PRESSO
MR RAPS
Weighted median
SNP effect on CONSTIPATION
SNP effect on T Ca
E
MR Test
Inverse variance weighted
MR Egger
MR PRESSO
MR RAPS
Weighted median
SNP effect on CONSTIPATION
SNP effect on EN Ca
F
MR Test
Inverse variance weighted
MR Egger
MR PRESSO
MR RAPS
Weighted median
SNP effect on CONSTIPATION
SNP effect on O Ca

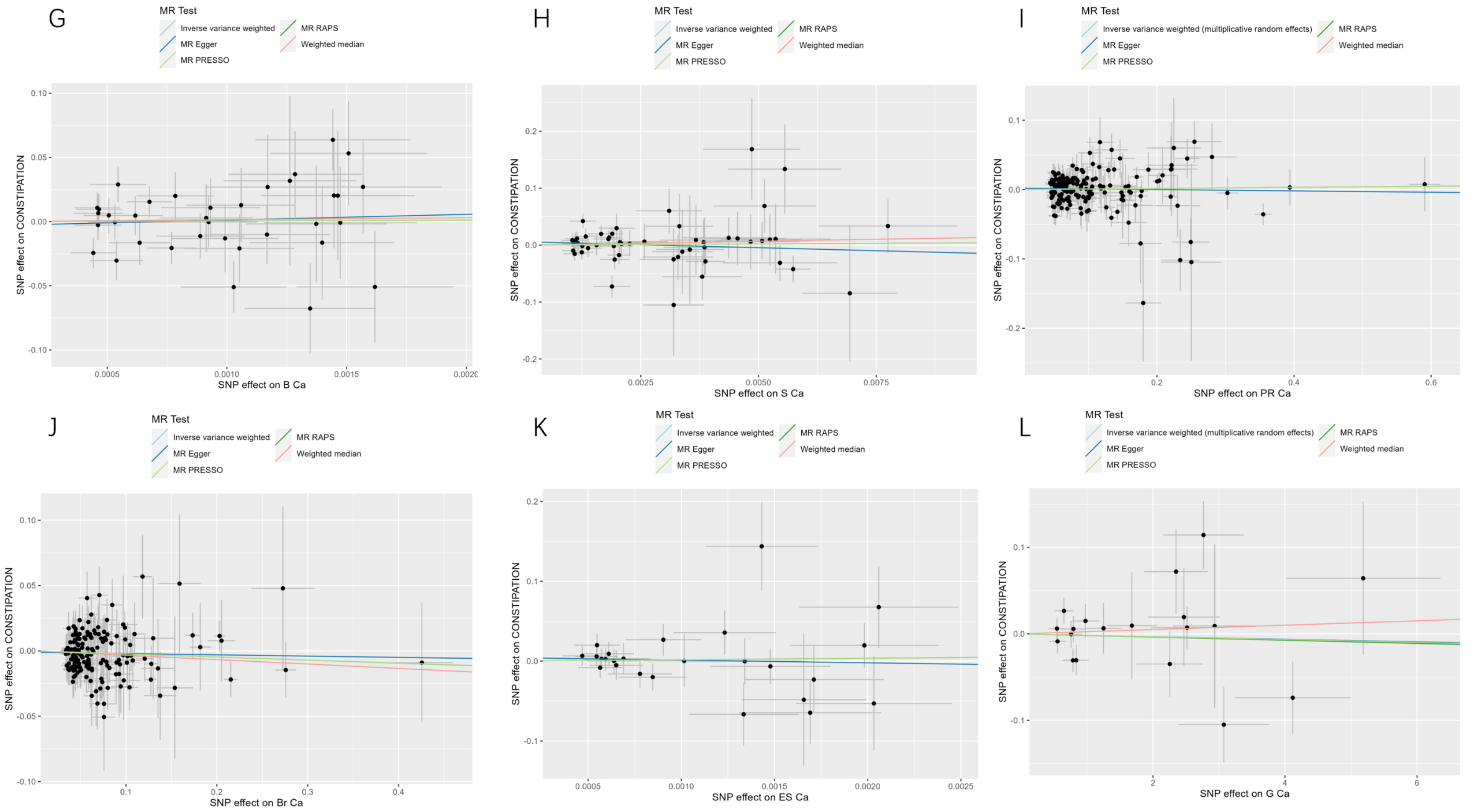
G
MR Test
Inverse variance weighted
MR Egger
MR PRESSO
MR RAPS
Weighted median
SNP effect on CONSTIPATION
SNP effect on B Ca
H
MR Test
Inverse variance weighted
MR Egger
MR PRESSO
MR RAPS
Weighted median
SNP effect on CONSTIPATION
SNP effect on S Ca
I
MR Test
Inverse variance weighted (multiplicative random effects)
MR Egger
MR PRESSO
MR RAPS
Weighted median
SNP effect on CONSTIPATION
SNP effect on PR Ca
J
MR Test
Inverse variance weighted
MR Egger
MR PRESSO
MR RAPS
Weighted median
SNP effect on CONSTIPATION
SNP effect on Br Ca
K
MR Test
Inverse variance weighted
MR Egger
MR PRESSO
MR RAPS
Weighted median
SNP effect on CONSTIPATION
SNP effect on ES Ca
L
MR Test
Inverse variance weighted (multiplicative random effects)
MR Egger
MR PRESSO
MR RAPS
Weighted median
SNP effect on CONSTIPATION
SNP effect on G Ca

M

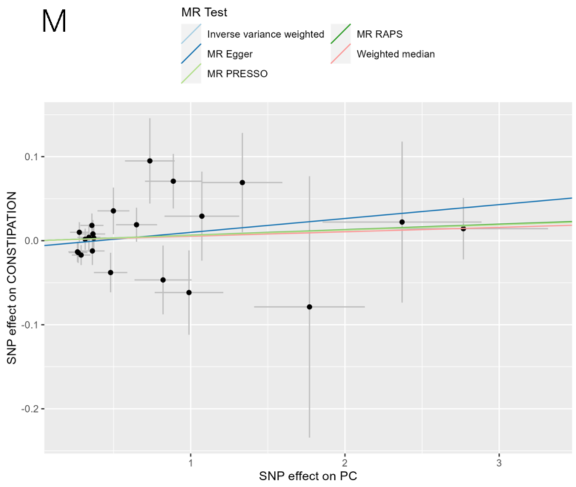


**Supplementary Figure 2-2 | Scatter plot of SNPs associated with (A) A) Bladder cancer; (B) Liver & bile duct cancer; (C) Cervical cancer; (D) Thyroid cancer; (E) Endometrial cancer; (F) Ovarian cancer(G) Brain cancer; (H) Skin melanoma; (I) Prostate cancer; (J) Breast cancer; (K) Esophageal cancer; (L) Gastric cancer; (M) Pancreatic cancer and risk on Constipation**

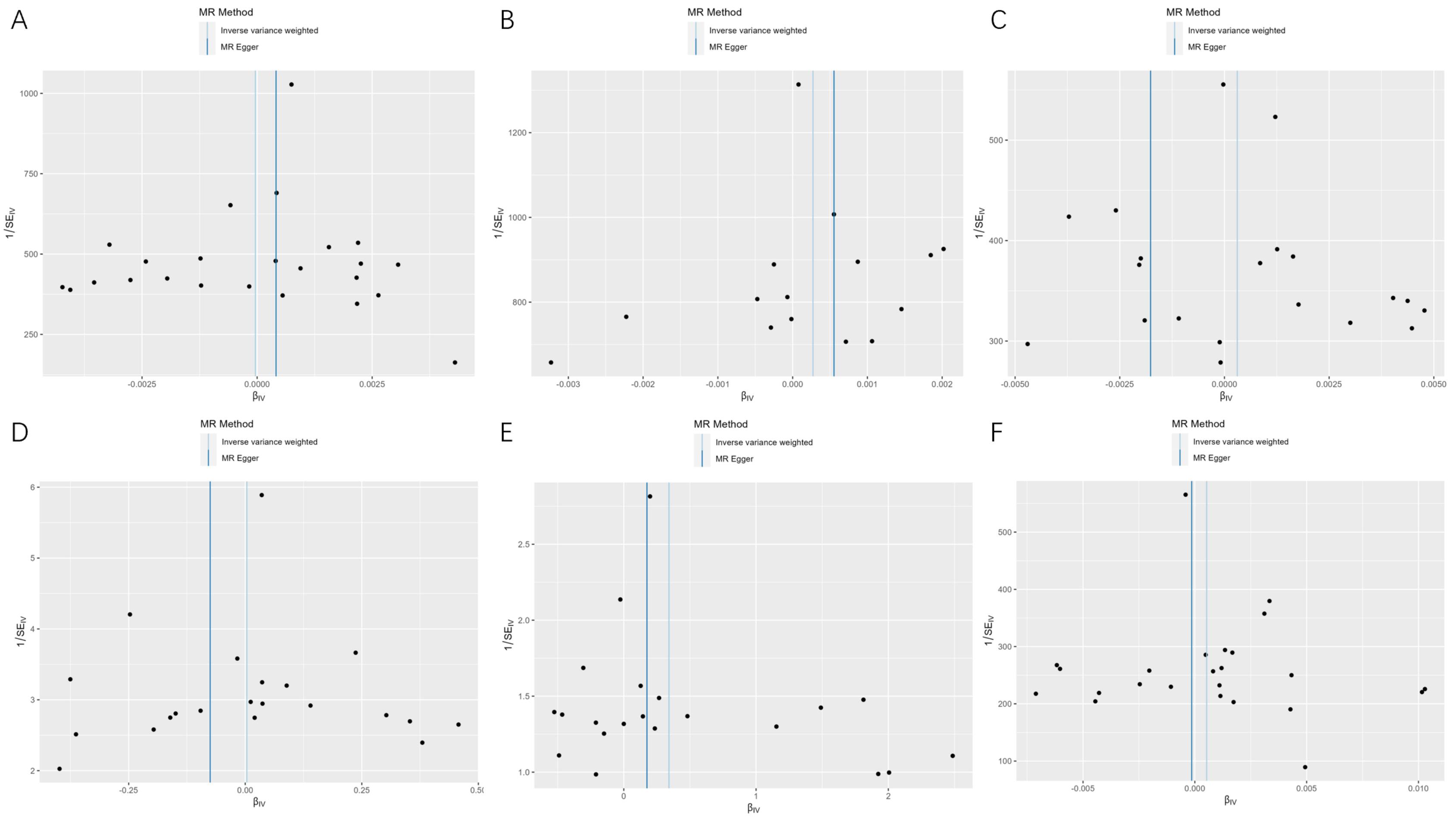
A
MR Method
Inverse variance weighted
MR Egger
1/SEIV
βIV
B
MR Method
Inverse variance weighted
MR Egger
1/SEIV
βIV
C
MR Method
Inverse variance weighted
MR Egger
1/SEIV
βIV
D
MR Method
Inverse variance weighted
MR Egger
1/SEIV
βIV
E
MR Method
Inverse variance weighted
MR Egger
1/SEIV
βIV
F
MR Method
Inverse variance weighted
MR Egger
1/SEIV
βIV

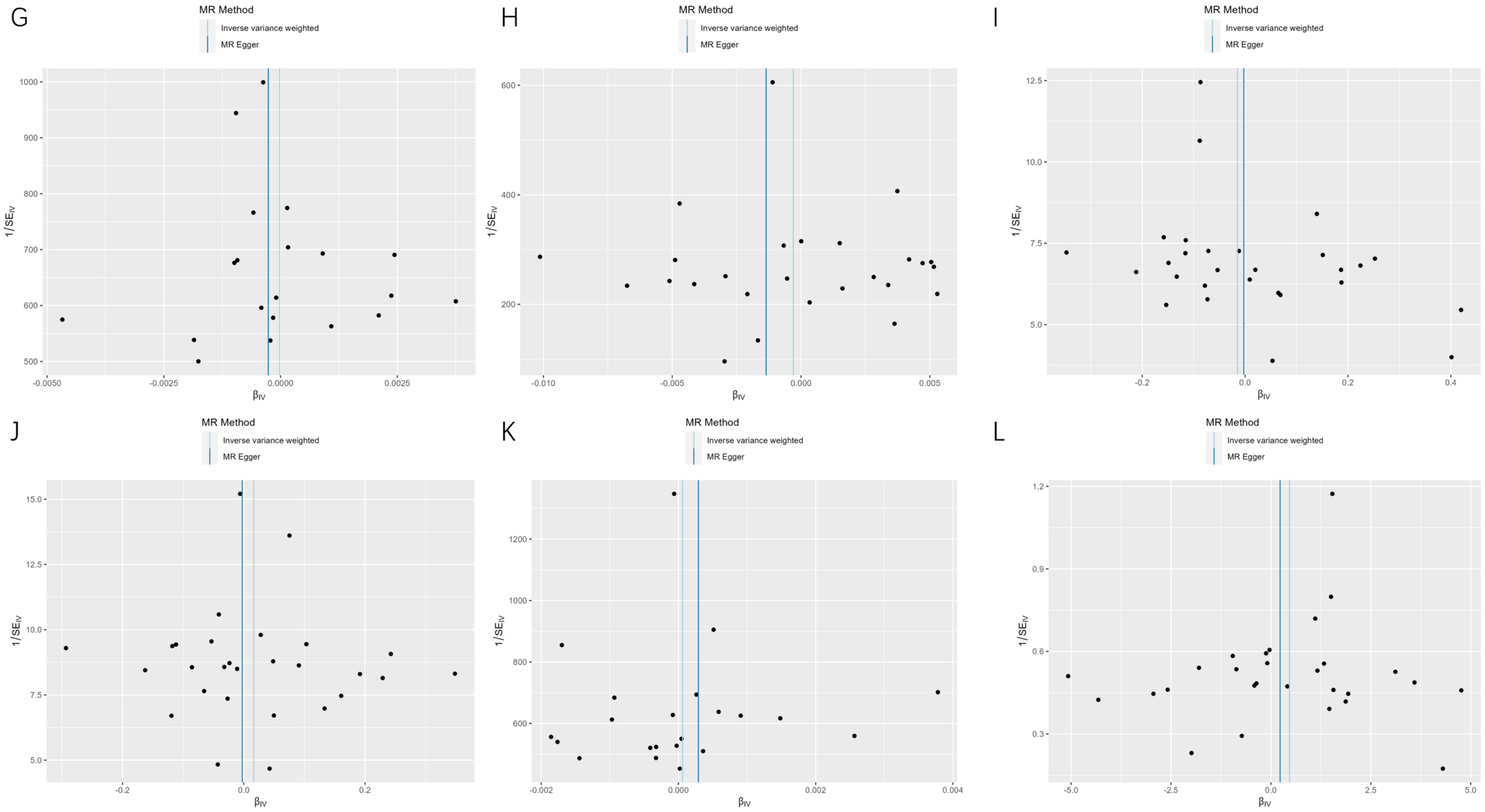
G
MR Method
Inverse variance weighted
MR Egger
1/SEIV
βIV
1000
900
800
700
600
500
-0.0050
-0.0025
0.0000
0.0025
H
MR Method
Inverse variance weighted
MR Egger
1/SEIV
βIV
600
400
200
-0.010
-0.005
0.000
0.005
I
MR Method
Inverse variance weighted
MR Egger
1/SEIV
βIV
12.5
10.0
7.5
5.0
-0.2
0.0
0.2
0.4
J
MR Method
Inverse variance weighted
MR Egger
1/SEIV
βIV
15.0
12.5
10.0
7.5
5.0
-0.2
0.0
0.2
K
MR Method
Inverse variance weighted
MR Egger
1/SEIV
βIV
1200
1000
800
600
-0.002
0.000
0.002
0.004
L
MR Method
Inverse variance weighted
MR Egger
1/SEIV
βIV
1.2
0.9
0.6
0.3
-5.0
-2.5
0.0
2.5
5.0

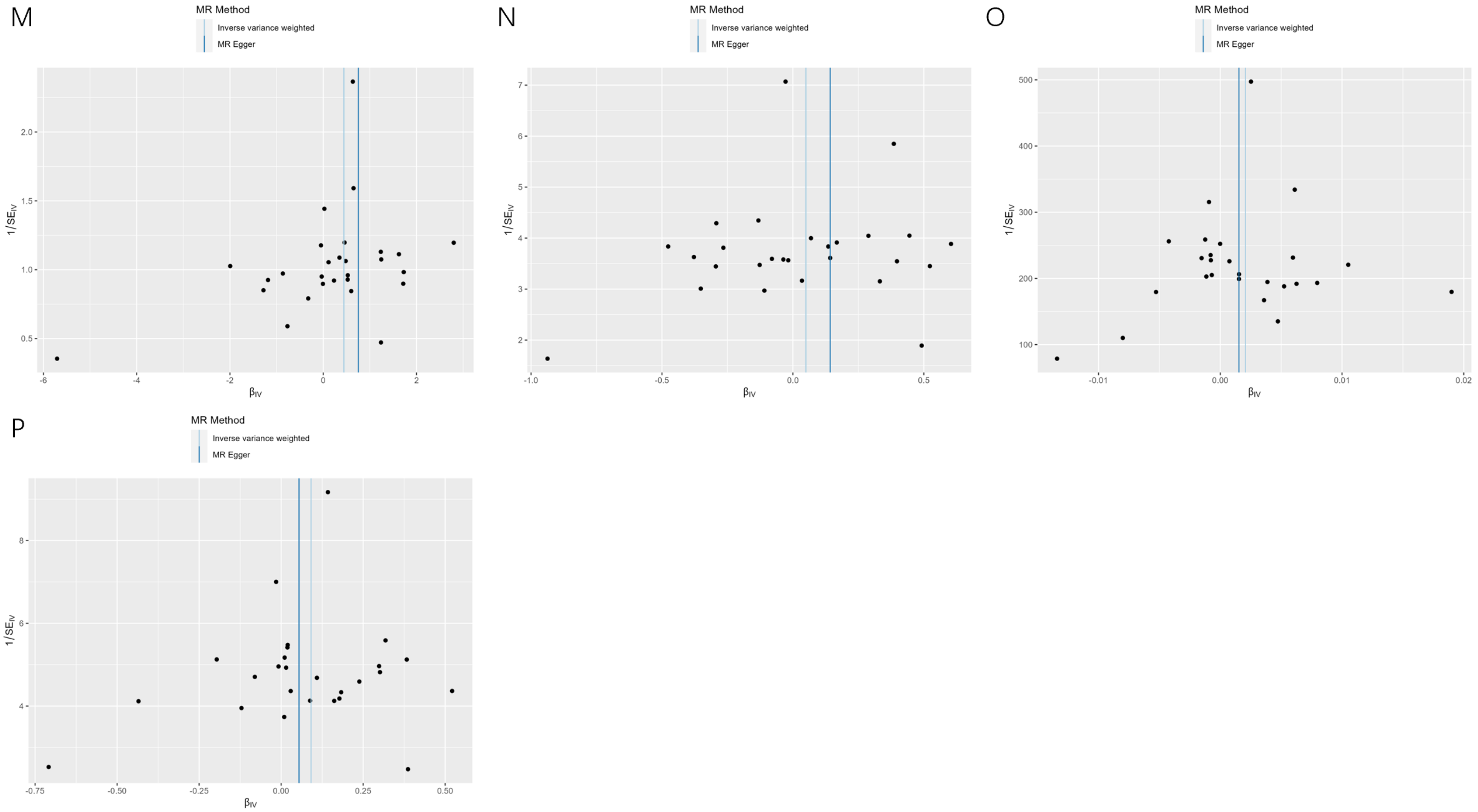


**Supplementary Figure 3-1 | Funnel plot of the relationship between the causal effect of Constipation on Cancer. Effect of Constipation on (A) Bladder cancer; (B) Liver & bile duct cancer; (C)Cervical cancer; (D) Thyroid cancer;(E) Oral cavity and pharyngeal cancer; (F) Ovarian cancer(G) Brain cancer; (H) Skin melanoma cancer; (I) Prostate cancer; (J) Breast cancer; (K) Esophageal cancer; (L) Gastric cancer; (M) Pancreatic cancer; (N) Endometrial cancer; (O) Colorectal cancer; (P) Lung cancer;**

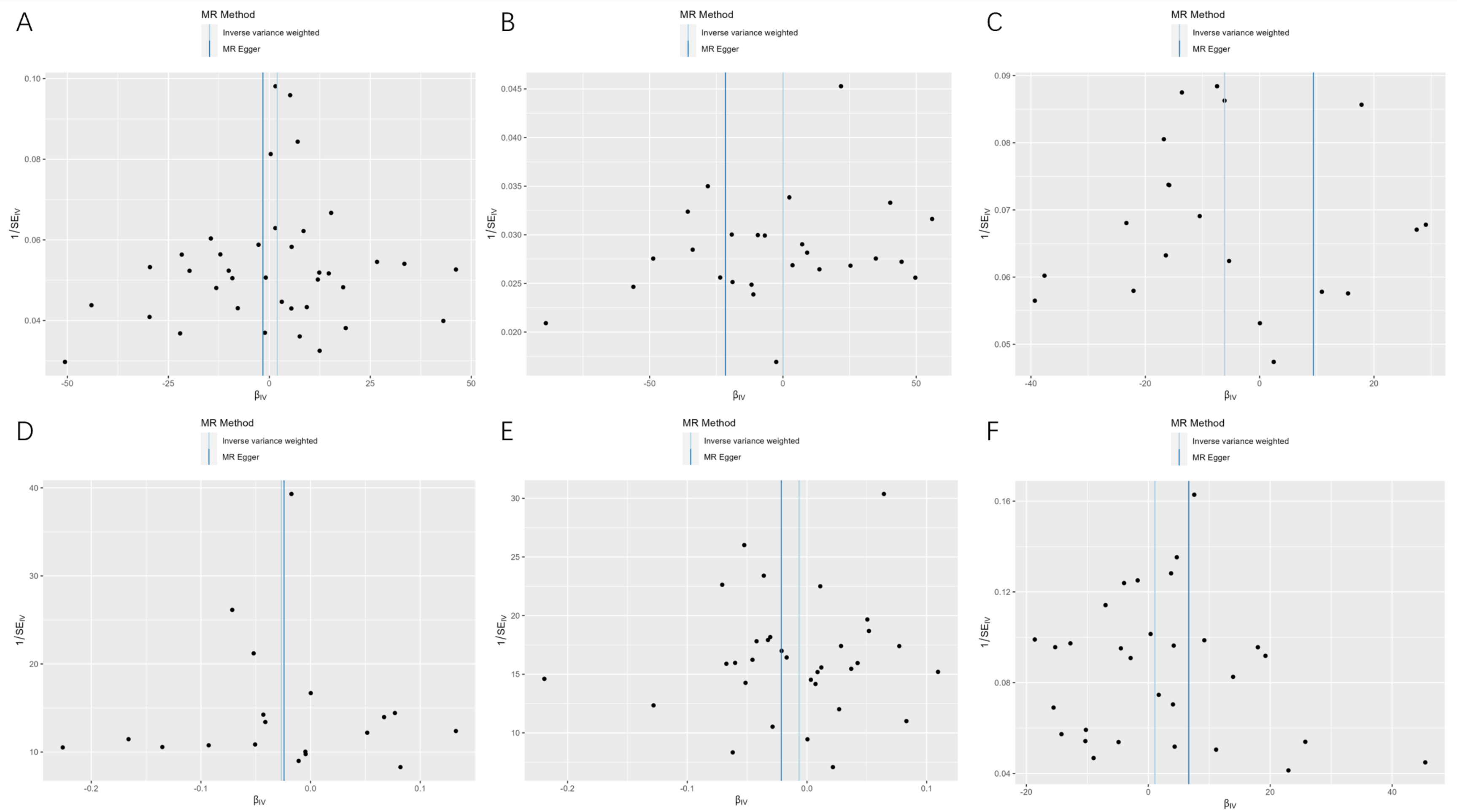
A
B
C
D
E
F
MR Method
Inverse variance weighted
MR Egger
1/SE_IV
β_IV

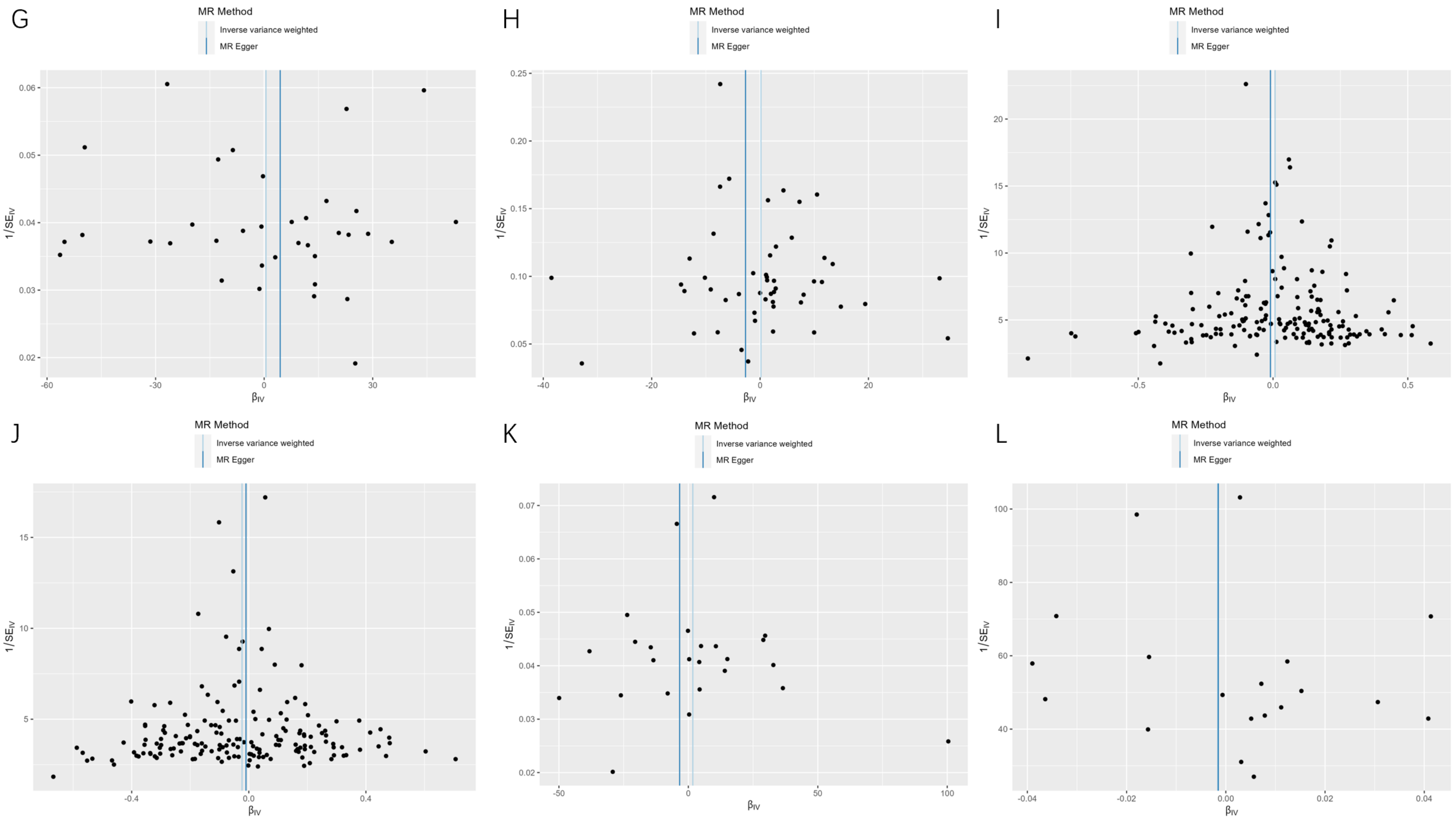

G
MR Method
Inverse variance weighted
MR Egger
1/SE$_{IV}$
0.06
0.05
0.04
0.03
0.02
-60
-30
0
30
β$_{IV}$
H
MR Method
Inverse variance weighted
MR Egger
1/SE$_{IV}$
0.25
0.20
0.15
0.10
0.05
-40
-20
0
20
β$_{IV}$
I
MR Method
Inverse variance weighted
MR Egger
1/SE$_{IV}$
20
15
10
5
-0.5
0.0
0.5
β$_{IV}$
J
MR Method
Inverse variance weighted
MR Egger
1/SE$_{IV}$
15
10
5
-0.4
0.0
0.4
β$_{IV}$
K
MR Method
Inverse variance weighted
MR Egger
1/SE$_{IV}$
0.07
0.06
0.05
0.04
0.03
0.02
-50
0
50
100
β$_{IV}$
L
MR Method
Inverse variance weighted
MR Egger
1/SE$_{IV}$
100
80
60
40
-0.04
-0.02
0.00
0.02
0.04
β$_{IV}$

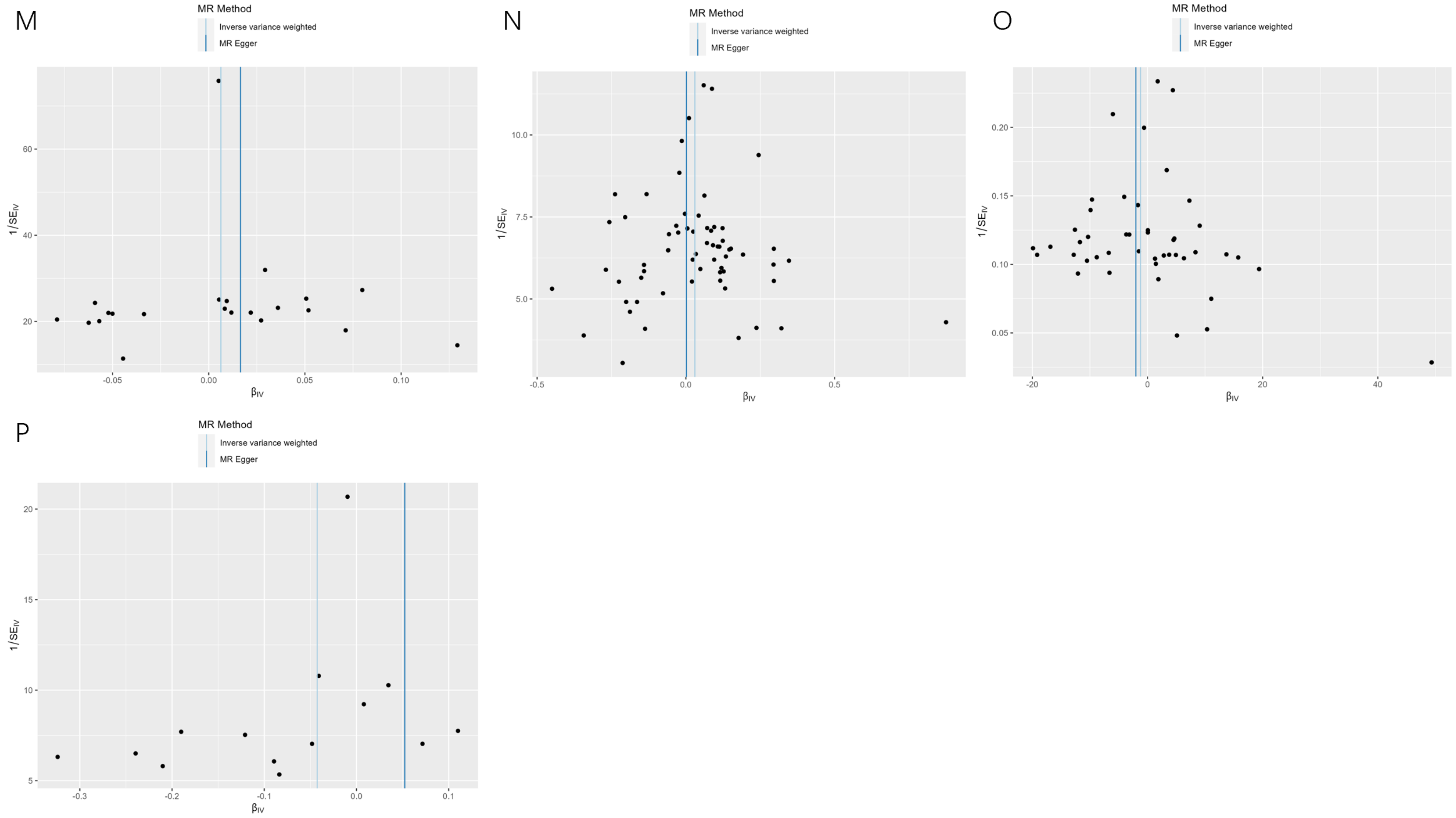


**Supplementary Figure 3-2 | Funnel plot of the relationship between the causal effect of Cancer on Constipation. Effect of (A) Bladder cancer; (B) Liver & bile duct cancer; (C)Cervical cancer; (D) Thyroid cancer;(E) Oral cavity and pharyngeal cancer; (F) Ovarian cancer(G) Brain cancer; (H) Skin melanoma cancer; (I) Prostate cancer; (J) Breast cancer; (K) Esophageal cancer; (L) Gastric cancer; (M) Pancreatic cancer; (N) Endometrial cancer; (O) Colorectal cancer; (P) Lung cancer on Constipation**

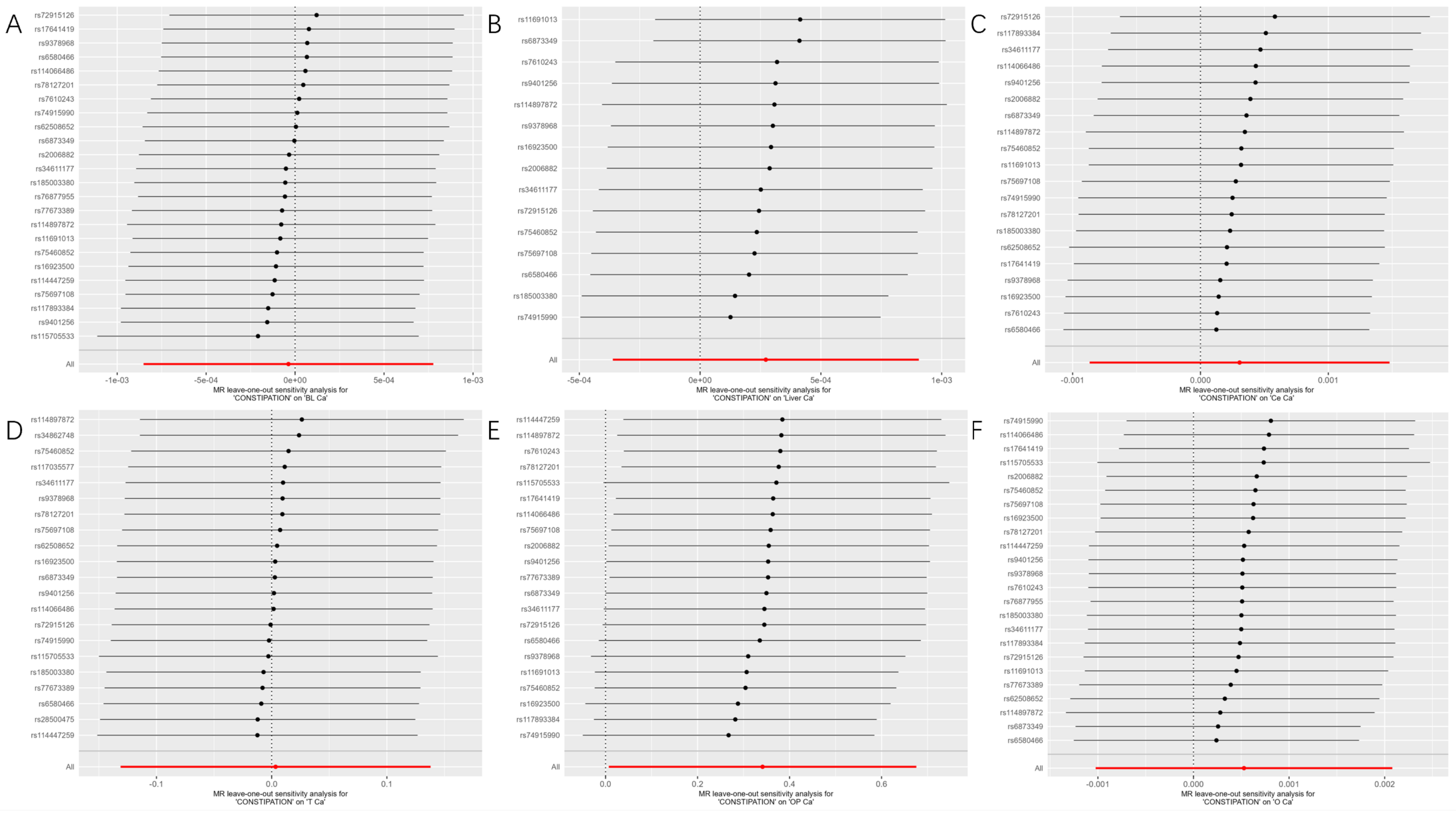
A
rs72915126
rs17641419
rs9378968
rs6580466
rs114066486
rs78127201
rs7610243
rs74915990
rs62508652
rs6873349
rs2006882
rs34611177
rs185003380
rs76877955
rs77673389
rs114897872
rs11691013
rs75460852
rs16923500
rs114447259
rs75697108
rs117893384
rs9401256
rs115705533
All
-1e-03
-5e-04
0e+00
5e-04
1e-03
MR leave-one-out sensitivity analysis for
'CONSTIPATION' on 'BL Ca'
B
rs11691013
rs6873349
rs7610243
rs9401256
rs114897872
rs9378968
rs16923500
rs2006882
rs34611177
rs72915126
rs75460852
rs75697108
rs6580466
rs185003380
rs74915990
All
-5e-04
0e+00
5e-04
1e-03
MR leave-one-out sensitivity analysis for
'CONSTIPATION' on 'Liver Ca'
C
rs72915126
rs117893384
rs34611177
rs114066486
rs9401256
rs2006882
rs6873349
rs114897872
rs75460852
rs11691013
rs75697108
rs74915990
rs78127201
rs185003380
rs62508652
rs17641419
rs9378968
rs16923500
rs7610243
rs6580466
All
-0.001
0.000
0.001
MR leave-one-out sensitivity analysis for
'CONSTIPATION' on 'Ce Ca'
D
rs114897872
rs34862748
rs75460852
rs117035577
rs34611177
rs9378968
rs78127201
rs75697108
rs62508652
rs16923500
rs6873349
rs9401256
rs114066486
rs72915126
rs74915990
rs115705533
rs185003380
rs77673389
rs6580466
rs28500475
rs114447259
All
-0.1
0.0
0.1
MR leave-one-out sensitivity analysis for
'CONSTIPATION' on 'T Ca'
E
rs114447259
rs114897872
rs7610243
rs78127201
rs115705533
rs17641419
rs114066486
rs75697108
rs2006882
rs9401256
rs77673389
rs6873349
rs34611177
rs72915126
rs6580466
rs9378968
rs11691013
rs75460852
rs16923500
rs117893384
rs74915990
All
0.0
0.2
0.4
0.6
MR leave-one-out sensitivity analysis for
'CONSTIPATION' on 'OP Ca'
F
rs74915990
rs114066486
rs17641419
rs115705533
rs2006882
rs75460852
rs75697108
rs16923500
rs78127201
rs114447259
rs9401256
rs9378968
rs7610243
rs76877955
rs185003380
rs34611177
rs117893384
rs72915126
rs11691013
rs77673389
rs62508652
rs114897872
rs6873349
rs6580466
All
-0.001
0.000
0.001
0.002
MR leave-one-out sensitivity analysis for
'CONSTIPATION' on 'O Ca'

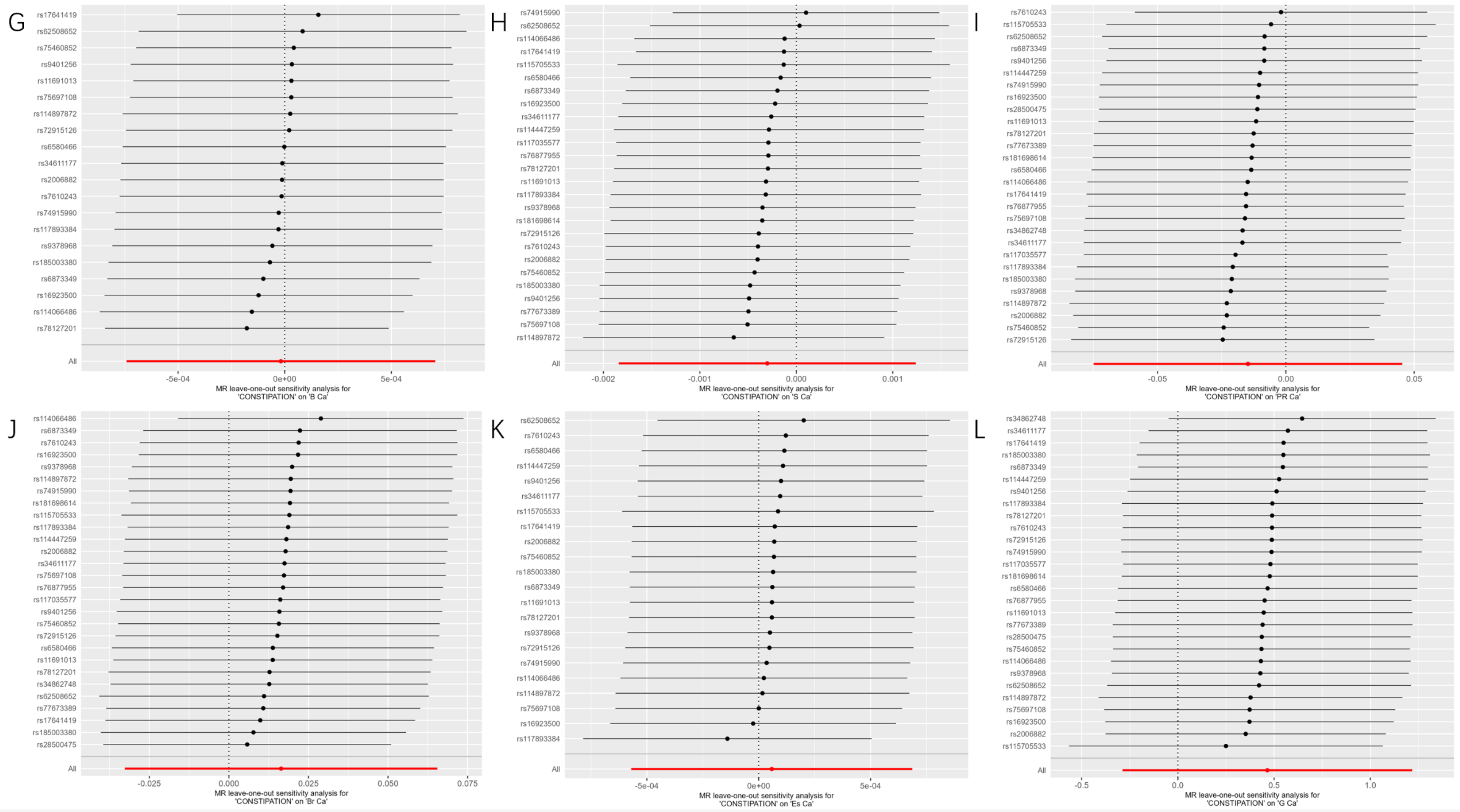
G
rs17641419
rs62508652
rs75460852
rs9401256
rs11691013
rs75697108
rs114897872
rs72915126
rs6580466
rs34611177
rs2006882
rs7610243
rs74915990
rs117893384
rs9378968
rs185003380
rs6873349
rs16923500
rs114066486
rs78127201
All
-5e-04
0e+00
5e-04
MR leave-one-out sensitivity analysis for
'CONSTIPATION' on 'B Ca'
H
rs74915990
rs62508652
rs114066486
rs17641419
rs115705533
rs6580466
rs6873349
rs16923500
rs34611177
rs114447259
rs117035577
rs76877955
rs78127201
rs11691013
rs117893384
rs9378968
rs181698614
rs72915126
rs7610243
rs2006882
rs75460852
rs185003380
rs9401256
rs77673389
rs75697108
rs114897872
All
-0.002
-0.001
0.000
0.001
MR leave-one-out sensitivity analysis for
'CONSTIPATION' on 'S Ca'
I
rs7610243
rs115705533
rs62508652
rs6873349
rs9401256
rs114447259
rs74915990
rs16923500
rs28500475
rs11691013
rs78127201
rs77673389
rs181698614
rs6580466
rs114066486
rs17641419
rs76877955
rs75697108
rs34862748
rs34611177
rs117035577
rs117893384
rs185003380
rs9378968
rs114897872
rs2006882
rs75460852
rs72915126
All
-0.05
0.00
0.05
MR leave-one-out sensitivity analysis for
'CONSTIPATION' on 'PR Ca'
J
rs114066486
rs6873349
rs7610243
rs16923500
rs9378968
rs114897872
rs74915990
rs181698614
rs115705533
rs117893384
rs114447259
rs2006882
rs34611177
rs75697108
rs76877955
rs117035577
rs9401256
rs75460852
rs72915126
rs6580466
rs11691013
rs78127201
rs34862748
rs62508652
rs77673389
rs17641419
rs185003380
rs28500475
All
-0.025
0.000
0.025
0.050
0.075
MR leave-one-out sensitivity analysis for
'CONSTIPATION' on 'Br Ca'
K
rs62508652
rs7610243
rs6580466
rs114447259
rs9401256
rs34611177
rs115705533
rs17641419
rs2006882
rs75460852
rs185003380
rs6873349
rs11691013
rs78127201
rs9378968
rs72915126
rs74915990
rs114066486
rs114897872
rs75697108
rs16923500
rs117893384
All
-5e-04
0e+00
5e-04
MR leave-one-out sensitivity analysis for
'CONSTIPATION' on 'Es Ca'
L
rs34862748
rs34611177
rs17641419
rs185003380
rs6873349
rs114447259
rs9401256
rs117893384
rs78127201
rs7610243
rs72915126
rs74915990
rs117035577
rs181698614
rs6580466
rs76877955
rs11691013
rs77673389
rs28500475
rs75460852
rs114066486
rs9378968
rs62508652
rs114897872
rs75697108
rs16923500
rs2006882
rs115705533
All
-0.5
0.0
0.5
1.0
MR leave-one-out sensitivity analysis for
'CONSTIPATION' on 'G Ca'

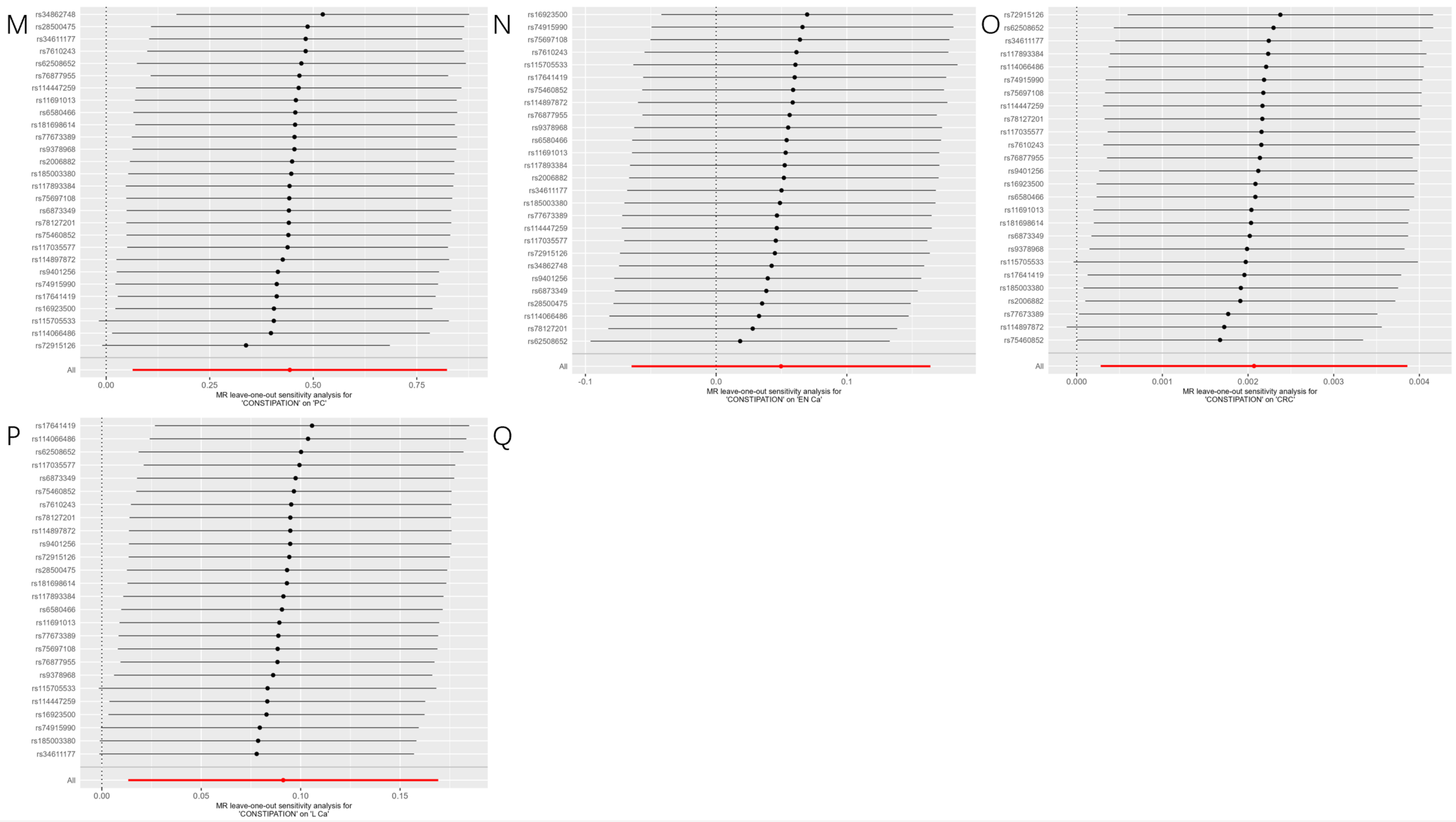


**Supplementary Figure 4-1 | Leave-one-out analysis result of SNPs associated with Constipation and risk on (A) Bladder cancer; (B) Liver & bile duct cancer; (C)Cervical cancer; (D) Thyroid cancer;(E) Oral cavity and pharyngeal cancer; (F) Ovarian cancer(G) Brain cancer; (H) Skin melanoma cancer; (I) Prostate cancer; (J) Breast cancer; (K) Esophageal cancer; (L) Gastric cancer; (M) Pancreatic cancer; (N) Endometrial cancer; (O) Colorectal cancer; (P) Lung cancer;**

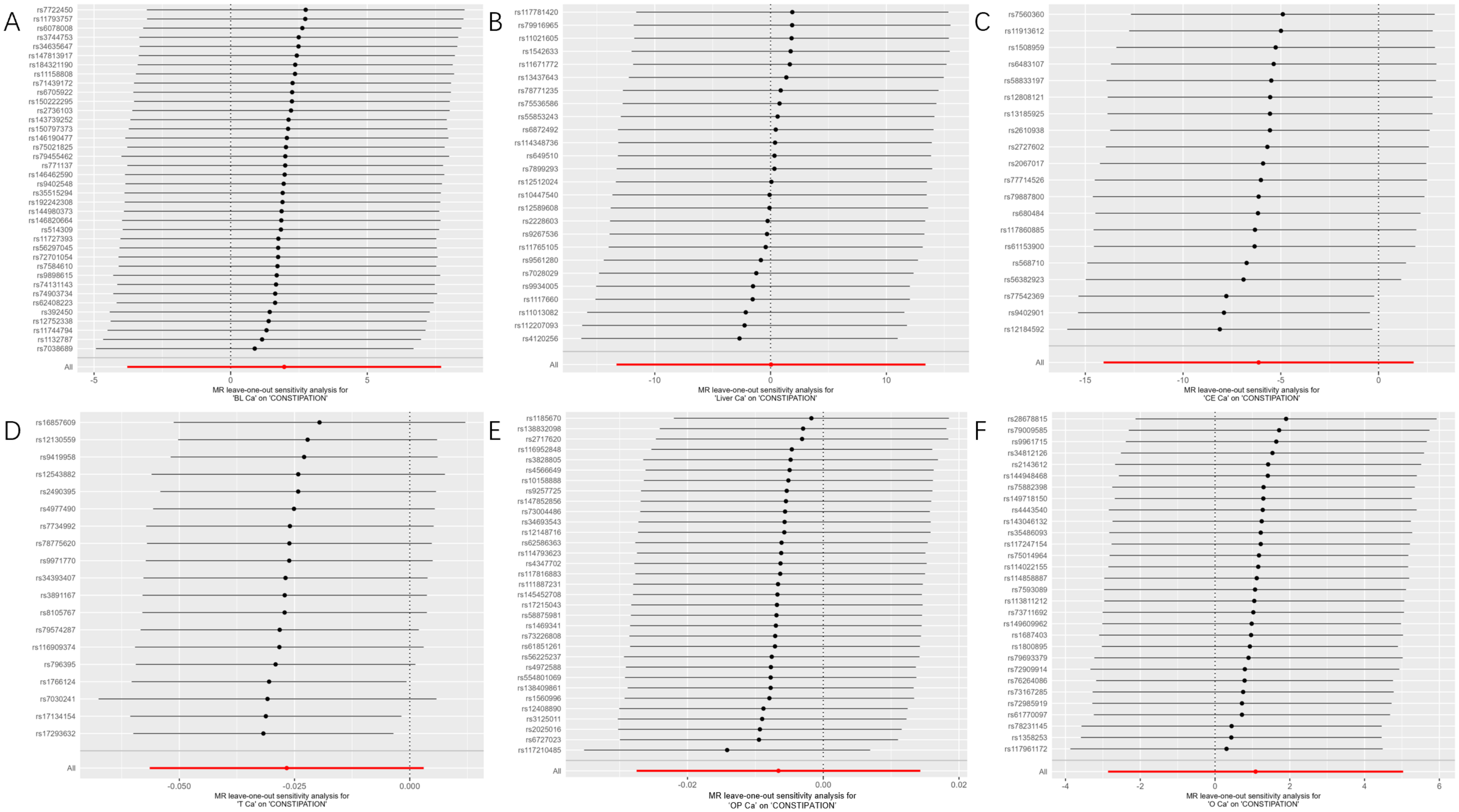
A
MR leave-one-out sensitivity analysis for
'BL Ca' on 'CONSTIPATION'
All
-5
0
5
B
MR leave-one-out sensitivity analysis for
'Liver Ca' on 'CONSTIPATION'
All
-10
0
10
C
MR leave-one-out sensitivity analysis for
'CE Ca' on 'CONSTIPATION'
All
-15
-10
-5
0
D
MR leave-one-out sensitivity analysis for
'T Ca' on 'CONSTIPATION'
All
-0.050
-0.025
0.000
E
MR leave-one-out sensitivity analysis for
'OP Ca' on 'CONSTIPATION'
All
-0.02
0.00
0.02
F
MR leave-one-out sensitivity analysis for
'O Ca' on 'CONSTIPATION'
All
-4
-2
0
2
4
6

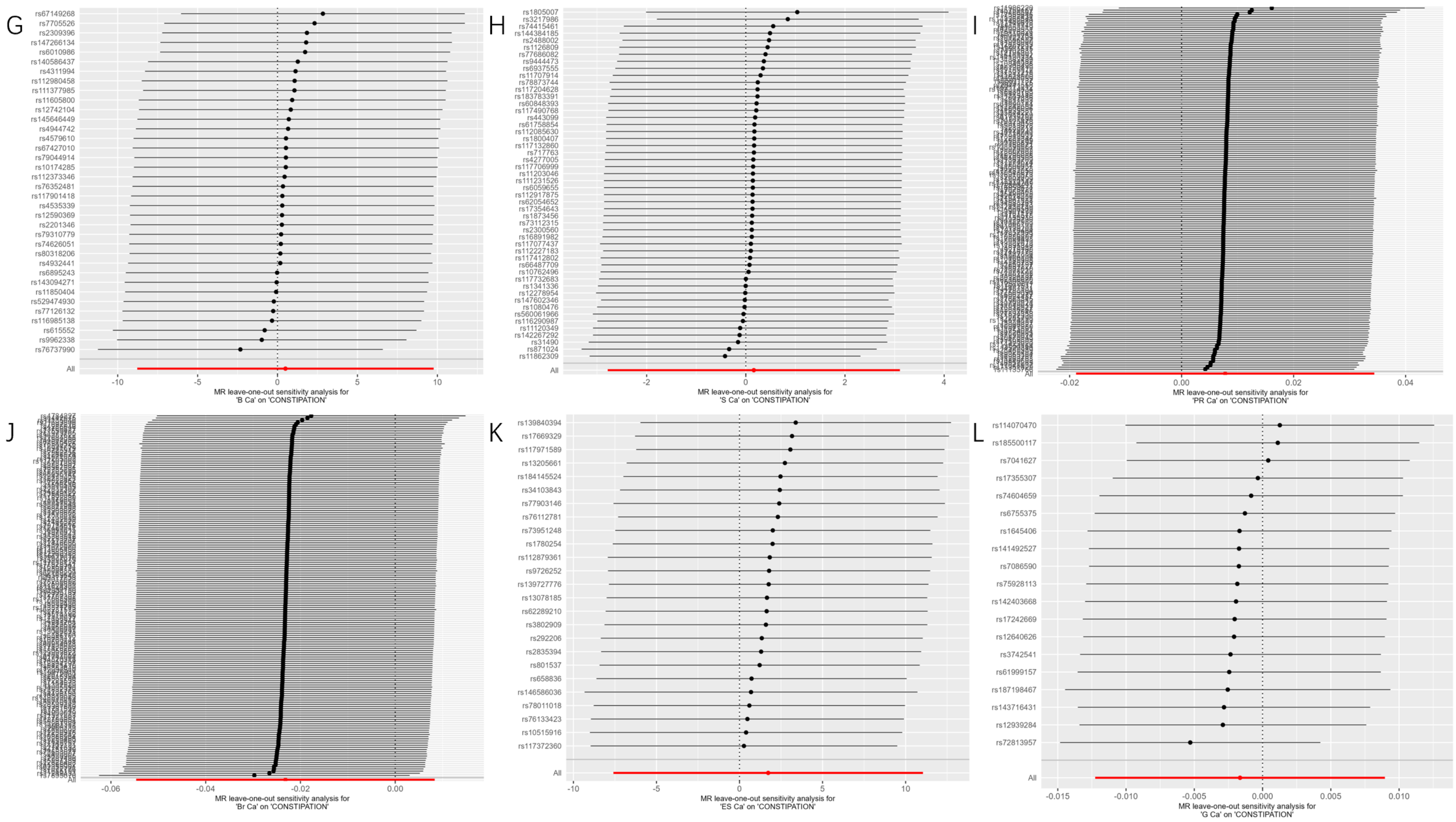
G
rs67149268
rs7705526
rs2309396
rs147266134
rs6010986
rs140586437
rs4311994
rs112980458
rs111377985
rs11605800
rs12742104
rs145646449
rs4944742
rs4579610
rs67427010
rs79044914
rs10174285
rs112373346
rs76352481
rs117901418
rs4535339
rs12590369
rs2201346
rs79310779
rs74626051
rs80318206
rs4932441
rs6895243
rs143094271
rs11850404
rs529474930
rs77126132
rs116985138
rs615552
rs9962338
rs76737990
All
-10
-5
0
5
10
MR leave-one-out sensitivity analysis for
'B Ca' on 'CONSTIPATION'
H
rs1805007
rs3217986
rs74415461
rs144384185
rs2488802
rs1126809
rs77686082
rs9444473
rs6937555
rs11707914
rs78873744
rs117204628
rs183783391
rs60848393
rs117490768
rs443099
rs61758854
rs112085630
rs1800407
rs117132860
rs717763
rs4277005
rs117706999
rs11203046
rs111231526
rs6059655
rs112917875
rs62054652
rs17354643
rs1873456
rs73112315
rs2300560
rs16891982
rs117077437
rs112227183
rs117412802
rs66487709
rs10762496
rs117732683
rs1341336
rs12278954
rs147602346
rs1080476
rs560061966
rs116290987
rs11120349
rs142267292
rs31490
rs871024
rs11862309
All
-2
0
2
4
MR leave-one-out sensitivity analysis for
'S Ca' on 'CONSTIPATION'
I
All
-0.02
0.00
0.02
0.04
MR leave-one-out sensitivity analysis for
'PR Ca' on 'CONSTIPATION'
J
All
-0.06
-0.04
-0.02
0.00
MR leave-one-out sensitivity analysis for
'Br Ca' on 'CONSTIPATION'
K
rs139840394
rs17669329
rs117971589
rs13205661
rs184145524
rs34103843
rs77903146
rs76112781
rs73951248
rs1780254
rs112879361
rs9726252
rs139727776
rs13078185
rs62289210
rs3802909
rs292206
rs2835394
rs801537
rs658836
rs146586036
rs78011018
rs76133423
rs10515916
rs117372360
All
-10
-5
0
5
10
MR leave-one-out sensitivity analysis for
'ES Ca' on 'CONSTIPATION'
L
rs114070470
rs185500117
rs7041627
rs17355307
rs74604659
rs6755375
rs1645406
rs141492527
rs7086590
rs75928113
rs142403668
rs17242669
rs12640626
rs3742541
rs61999157
rs187198467
rs143716431
rs12939284
rs72813957
All
-0.015
-0.010
-0.005
0.000
0.005
0.010
MR leave-one-out sensitivity analysis for
'G Ca' on 'CONSTIPATION'

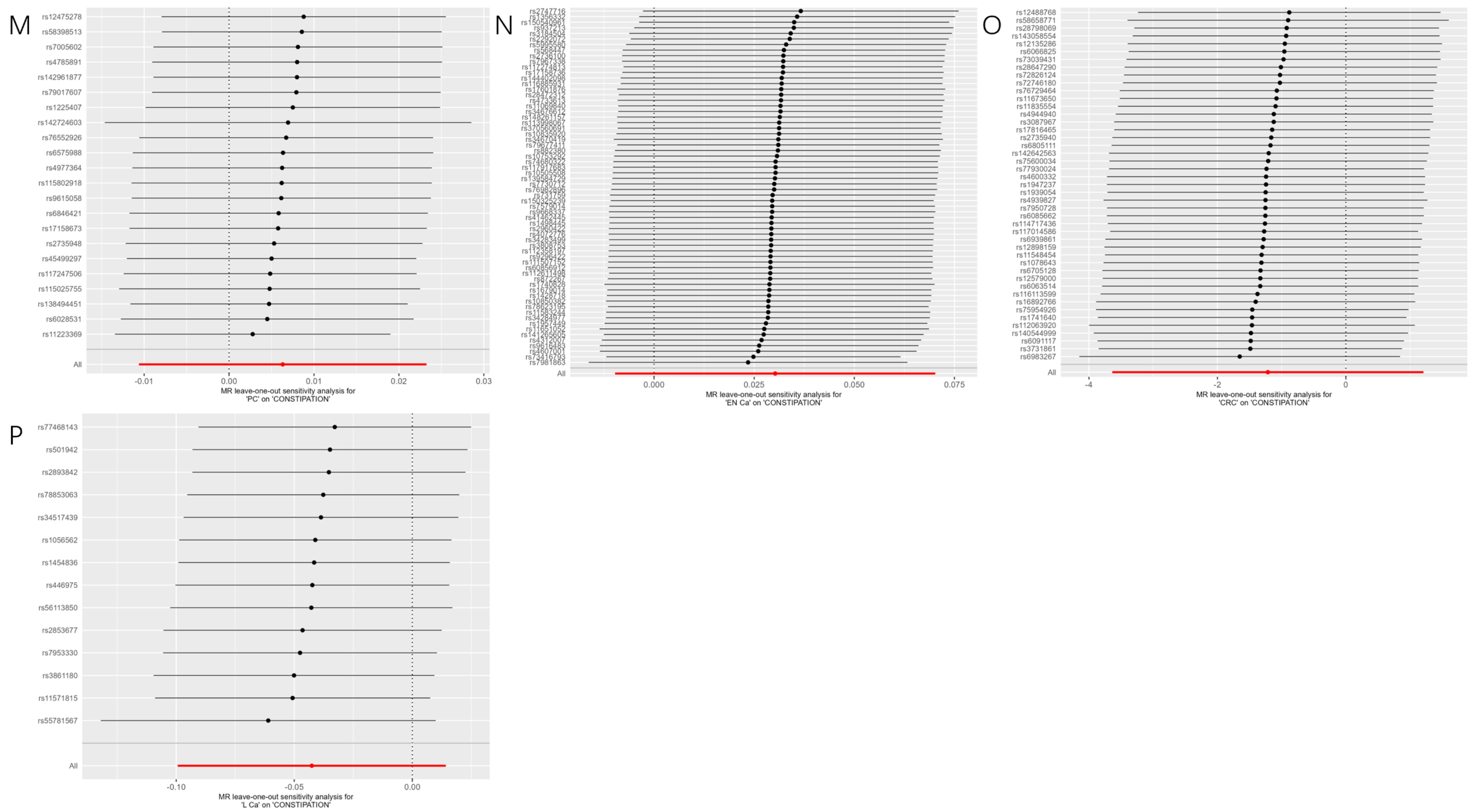


**Supplementary Figure 4-2 | Leave-one-out analysis result of SNPs associated with(A) Bladder cancer; (B) Liver & bile duct cancer; (C)Cervical cancer; (D) Thyroid cancer;(E) Oral cavity and pharyngeal cancer; (F) Ovarian cancer(G) Brain cancer; (H) Skin melanoma cancer; (I) Prostate cancer; (J) Breast cancer; (K) Esophageal cancer; (L) Gastric cancer; (M) Pancreatic cancer; (N) Endometrial cancer; (O) Colorectal cancer; (P) Lung cancer and risk on Constipation**
